# Optical and Structural Properties of Si Nanocrystals in SiO_2_ Films

**DOI:** 10.3390/nano5020614

**Published:** 2015-04-22

**Authors:** Timur Nikitin, Leonid Khriachtchev

**Affiliations:** Department of Chemistry, University of Helsinki, P.O. Box 55, FI-00014 Helsinki, Finland; E-Mail: timnik@eumx.net

**Keywords:** Si nanocrystal (Si-nc), SiO_2_ film, Raman spectroscopy, photoluminescence, laser annealing

## Abstract

Optical and structural properties of Si nanocrystals (Si-nc) in silica films are described. For the SiO*_x_* (*x* < 2) films annealed above 1000 °C, the Raman signal of Si-nc and the absorption coefficient are proportional to the amount of elemental Si detected by X-ray photoelectron spectroscopy. A good agreement is found between the measured refractive index and the value estimated by using the effective-medium approximation. The extinction coefficient of elemental Si is found to be between the values of crystalline and amorphous Si. Thermal annealing increases the degree of Si crystallization; however, the crystallization and the Si–SiO_2_ phase separation are not complete after annealing at 1200 °C. The 1.5-eV PL quantum yield increases as the amount of elemental Si decreases; thus, this PL is probably not directly from Si-nc responsible for absorption and detected by Raman spectroscopy. Continuous-wave laser light can produce very high temperatures in the free-standing films, which changes their structural and optical properties. For relatively large laser spots, the center of the laser-annealed area is very transparent and consists of amorphous SiO_2_. Large Si-nc (up to ~300 nm in diameter) are observed in the ring around the central region. These Si-nc lead to high absorption and they are typically under compressive stress, which is connected with their formation from the liquid phase. By using strongly focused laser beams, the structural changes in the free-standing films can be made in submicron areas.

## 1. Introduction

Many limitations of modern electronic devices may be overcome by implementing photonics into electronics [1,2,3,4]. Integration of Si-based photonics with CMOS technology is a promising approach because it gives the possibility to merge electronics and photonics in the same chip [5,6,7,8,9]. Many optical functions such as, for example, light sources, amplifiers, waveguides, modulators, memory, and detectors should be achieved in order to fulfill this integration. Realization of a true monolithically fabricated injection Si laser with a small size is a particularly challenging task. The fundamental problem is the low light-emitting efficiency of bulk silicon due to its indirect band gap (~1.1 eV), which leads to long radiative times (~ms) and therefore, mostly non-radiative recombination of the excited carriers. The efficiency of the light emission can be enhanced by increasing the overlap of the wave functions of the electron and hole via, for example, spatial confinement [10,11,12,13]. The unusual properties of Si structures are observed when the size is less than the free exciton Bohr radius of 4.3 nm in bulk Si. This quantum confinement (QC) effect leads to the following main changes in the material properties: (i) an increase of the radiative probability due to delocalization of the wave functions in the momentum space, which increases the electron-hole wave function overlap and (ii) a shift in the emission wavelength to the visible region due to an increase of the band gap, which is controlled by the Si nanostructure size. Moreover, the number of sites within the volume of Si nanostructures, where non-radiative recombination can occur, decreases considerably. It should be mentioned that understanding electrical transport mechanisms in the systems considered below is very important for many applications [14]; however, this topic is outside the scope of the present review.

Porous Si is the first example of a Si-based material with enhanced luminescence efficiency. Strong room-temperature photoluminescence (PL) in visible spectral region from this material was discovered by Leigh Canham in 1990 [15]. Visible luminescence ranging from green to red in color was soon reported by Canham *et al.* [16] for other porous-Si samples and by other researchers [17,18] and ascribed to the quantum effects in Si structures with a size of ~3 nm. The blue shift of the PL and optical absorption with the increasing porosity (decreasing Si nanocluster sizes) provided the first important evidence that the QC plays an important role in light emission from porous Si. This blue shift is a result of the band gap expansion controlled by the size of Si nanostructures [13,19].

It was soon understood that oxidation of small Si crystallites has a great influence on the light emission [20,21]. The study by Wolkin *et al.* of oxidized porous Si has shown that the light-emitting centers can involve the Si=O covalent bonds located on the crystallite surface [22]. For oxygen-passivated Si crystallites, a stabilized electronic surface state is formed on a Si=O covalent bond and various recombination mechanisms can operate depending on the crystallite size. For larger sizes (>3 nm), recombination occurs via free excitons since the band gap is not wide enough to stabilize the Si=O surface state. For intermediate sizes (~2.5 nm), recombination involves a trapped electron localized on the Si atom of the Si=O bond and a free hole. For smaller sizes (<2 nm), recombination occurs via trapped excitons.

However, QC has been demonstrated for a number of Si nanomaterials. In contrast with oxygen passivation, for hydrogen-passivated porous Si, recombination occurs via free exciton states for all crystallite sizes and follows the expected QC behavior [22]. Ledoux *et al*. studied Si nanocrystals (Si-nc) prepared by pulsed CO_2_ laser pyrolysis of silane in a gas flow reactor and deposited on a substrate [23]. It was observed that the PL band was blue-shifted as the size of Si-nc decreased from 8 to 2.5 nm. This dependence clearly follows the QC model. The QC mechanism operates also for alkane-terminated Si-nc. Hannah *et al*. [24] convincingly demonstrated using pressure-dependent PL studies that the PL arises from the core-states of Si-nc with indirect-gap transitions. The same PL mechanism has also been reported to operate for Si-nc in silicon-nitride films [25,26].

Another attractive Si-based material, which is the focus of the present article, is composed of Si-nc embedded in a SiO_2_ matrix. This material is chemically and mechanically more stable compared to porous Si and also emits light in the visible region. Si-nc in silica films can be prepared by various methods such as, for example, molecular beam deposition (MBD) [27,28], Si-ion implantation [29,30,31,32], sputtering [33,34,35,36,37,38,39], plasma enhanced chemical vapor deposition (PECVD) [33,34,40,41,42], low pressure chemical vapor deposition with subsequent thermal oxidation [43,44,45], reactive evaporation of SiO powder in oxygen atmosphere [46], and electron beam deposition [47]. Two types of architectures are most commonly prepared: (i) Si-rich silicon oxide SiO*_x_* (*x* < 2) films [31,33,40,48,49] and (ii) SiO*_x_*/SiO_2_ [37,46,47,50,51,52,53] and Si/SiO_2_ [27,28,35,54,55,56,57] superlattices (SLs). Si-nc are formed in these materials by furnace annealing above 1000 °C. Studies of Si-nc in SiO_2_ have been particularly stimulated by the observation of optical gain in this material [58,59]. In addition to PL, electroluminescence in visible region from porous Si and from Si-nc in SiO_2_ has been reported [60,61].

The origin of light emission from Si-nc in a SiO_2_ matrix is still controversial. It seems that the conclusions on the mechanism of the light emission from oxidized porous Si [20,21,22,62] are relevant to Si-nc in SiO_2_. Similarly, the PL of Si-nc (sizes > 3 nm) in SiO_2_ can be ascribed to the QC effect [63,64,65,66]. On the other hand, the importance of the Si-nc/SiO_2_ interface in the light-emitting properties has been stressed [31,35,67,68] and, in particular, the defect origin of the PL has been discussed [27,29,31,69,70,71]. Godefroo *et al*. have convincingly demonstrated that defects are the dominant source of the 1.5-eV PL [52]. In their experiment, an amorphous SiO/SiO_2_ SL grown by reactive evaporation of SiO powders in an oxygen atmosphere was thermally annealed at 1100 °C for 1 h under N_2_ atmosphere to produce Si-nc. The authors could switch the PL mechanism between the QC and defect mechanisms by passivation with hydrogen and ultraviolet illumination, respectively.

Optoelectronic applications of Si-based materials require knowledge of their optical and structural properties. For SiO*_x_* (*x* < 2) films prepared by PECVD, the energy-filtered transmission electron microscopy (EFTEM) show that the Si–SiO_2_ phase separation starts after annealing at 900 °C for 1 h under N_2_ atmosphere when Si clusters become visible in an oxide matrix [72]. The dark-field (DF) transmission electron microscopy (TEM) technique reliably shows that the Si clusters are amorphous for annealing temperatures of 900–1000 °C, and their crystallization begins at 1100 °C. Higher annealing temperatures promote further crystallization of the Si clusters. The Si-nc size as a function of the annealing temperature and Si content has been studied for samples prepared by different deposition methods. For samples prepared by PECVD, the TEM results indicate that the Si-nc sizes increase with the increasing annealing temperature for a given Si content and with the Si content for a given annealing temperature [40]. The increase of the Si-nc sizes with the annealing temperature is also observed for SiO*_x_* films grown by magnetron sputtering [37]. For SiO*_x_* films prepared by ion implantation, the nucleation and pure growth stages of the nanocrystal population are almost over after 1 min of annealing at 1100 °C in N_2_ [31]. For longer annealing periods, the sizes of Si-nc increase due to coalescence or Ostwald ripening process [31,73].

Several methods have been employed to study optical constants of different materials containing Si-nc. These methods include, for example, optical ellipsometry [74,75,76] and *m*-line measurements [77,78,79]. The analysis of the optical properties often uses the Bruggeman effective-medium approximation [80,81,82]. The Tauc-Lorentz model provides optical constants of Si-nc deposited on a substrate [82]. The results show that the optical properties of Si-nc are quite different from those of amorphous and crystalline Si. It has been found that at a given wavelength, the refractive index of SiO*_x_* films increases for larger Si-nc for Si-ion implanted samples [74] and for larger Si content for PCVD samples [75]. Chen *et al.* have developed a quantitative approach of obtaining depth profiles of the optical constants [81]. Moreno *et al.* report that the refractive index of Si-nc is lower than that of amorphous and crystalline bulk Si [80]. They also conclude that the refractive index is rather independent of the Si clusters sizes (3.6–4.6 nm) and is mostly affected by the degree of crystallinity. For SiO*_x_*/SiO_2_ SLs prepared by magnetron sputtering, negative optical birefringence (~1%) was observed, originating from the periodical set of parallel planes of two different materials constituting the SL [77,78]. At a given wavelength, the absorption coefficient of annealed SiO*_x_* films prepared by magnetron sputtering and PECVD increases with the Si content [33,76,83]. The increase of the Si-nc mean diameter leads to an increase of absorption in porous Si [84] and in SiO*_x_* films prepared by ion implantation [80]. For a fixed Si content, the absorption coefficient of films prepared by magnetron sputtering and PECVD decreases with the increasing annealing temperature, which is due the amorphous-to-crystalline transition of Si in the samples [33].

Another method of measuring the optical properties of SiO*_x_* films is based on the PL-filtering effect. A silicon oxide layer containing Si-nc on a silica substrate forms a planar waveguide. Spectral filtering of the PL occurs when the PL is detected from the waveguide edge along the film surface. This effect has been found for Si/SiO_2_ SLs [57] and for SiO*_x_* films (*x* < 2) on silica substrate [79,85,86,87]. The detailed description of this effect is presented later.

The Tauc relation [88] allows one to estimate the band gap of SiO*_x_* films containing Si-nc [31,33,36,44,74,89,90]. A study of Si-nc deposited on a silica substrate shows that the electronic band structure of Si-nc is quite different from that of bulk silicon and that the band gap increases as the Si-nc size decreases [82]. The latter observation is explained by the QC effect. The increase of the band gap for smaller Si-nc is also observed for implanted SiO*_x_* samples [31,74]. The absorption threshold is blue-shifted for smaller Si-nc, which indicates the QC effect on the Si-nc band gap [33,36,84,90,91].

Laser annealing is an interesting approach to change the structural and optical properties of Si-based materials. The heating effect of pulsed radiation is especially strong [92]. However, it can also be substantial for continuous-wave (CW) irradiation of free-standing porous Si films [93]. A similar heating effect of CW laser radiation is observed in free-standing SiO*_x_* films leading to a strong increase of the Raman signal of Si-nc [56,94,95,96]. Si-nc prepared by laser annealing show high compressive stress (~3 GPa), which is evidenced by an up-shift of the Raman band [56,96]. The compressive stress is formed in a solid SiO_2_ matrix when the volume of Si-nc suddenly increases after its crystallization from the liquid phase. The high stress can be relaxed by irradiating the stressed Si-nc with lower laser power [56]. Free-standing films are used to obtain the above described laser-induced effects because for films on substrates the laser-induced heat is reduced due to thermal flux to substrate [97]; however, it is still observable [98].

In agreement with the experimental observations, the theoretical simulations of isolated hydrogen-passivated Si nanostructures show that the band gap and the PL peak position change according to QC [13,19]. The behavior of these parameters is different for oxidized Si-nc [99]. Theoretical studies highlight the importance of the interface region between Si-nc and a SiO_2_ matrix for the light-emitting properties [100,101]. The absorption edge tends to be red-shifted due to the formation of the Si=O or Si–O–Si bonds at the Si/SiO_2_ interface [102,103,104]. The presence of oxygen atoms bonded to the surface of Si-nc as well as deformation of Si-nc are found to influence the optical band gap [105]. Luppi *et al*. have considered small Si-nc (up to 1 nm in diameter) by applying *ab initio* calculations [106]. In the case of the Si–O–Si bridge bond at the cluster surface, an emission peak at about 1.5 eV is obtained. In accord, the PL in this region is often observed experimentally from silica films containing Si-nc. The calculated emission peak is red-shifted with respect to the absorption, which is also in agreement with the experimentally observed Stokes shifts between the absorption and PL spectra.

Theoretical calculations show that the surrounding matrix can produce some strain on Si-nc [107,108,109], and this strain depends on the oxidation degree [110,111]. The magnitude of strain affects the band gap, which shows an oscillating behavior with the Si-nc size, not strictly following the QC rule [110]. Moreover, the disorder of nanoclusters has a large effect on their optoelectronic properties [112,113]. In particular, amorphization reduces the optical band gap and increases the absorption strength in the visible range.

Guerra and Ossicini have calculated the recombination rates for different classes of Si-nc in the diameter range of 0.2–1.5 nm [114]. The authors also consider different conditions of passivation, strain, and symmetry in order to find the best conditions of radiative emission. As a result, they have found that the smallest, highly oxidized, crystalline clusters are the most optically active Si/SiO_2_ structures.

## 2. Results and Discussion

### 2.1. Furnace-Annealed Films

In this section, we describe the properties of SiO*_x_* (*x* < 2) films on silica substrates for different annealing temperatures (400–1200 °C) and Si contents (*x* from ~1.3 to ~1.98) [115,116,117,118]. The SiO*_x_* films (thickness from ~1.5 to ~2.5 μm) are deposited on silica substrates by MBD. The MBD samples are compared with samples prepared by ion implantation [118].

#### 2.1.1. Correlation between Optical and Structural Properties

**Figure 1:**
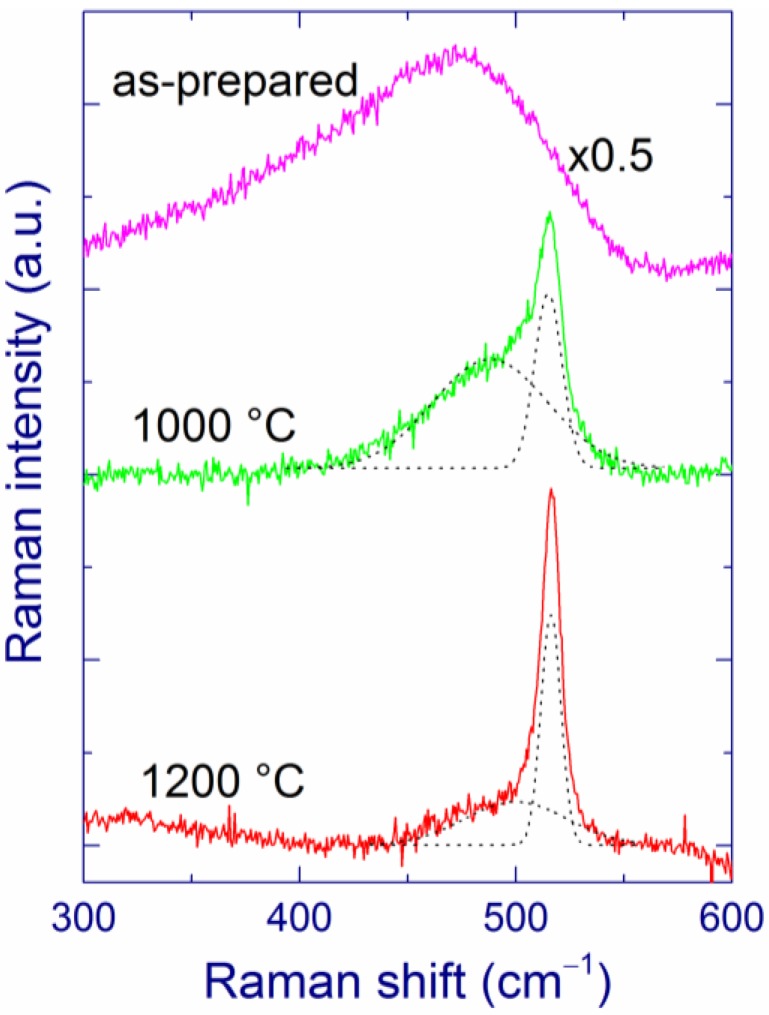
Raman spectra of a SiO_1.75_ film as-prepared and annealed at 1000 and 1200 °C (fitted by two Gaussians). The spectra are vertically shifted for better presentation. The Raman spectra are measured with laser intensity of ~10^3^ W cm^–2^ at the sample. Reproduced with permission from [116]. Copyright 2012, AIP Publishing LLC.

The as-prepared MBD films (annealed at 400 °C for better mechanical stability) with substantial Si content show a broad Raman band at ~470 cm^–1^, which is characteristic of amorphous Si (Figure 1) [27,48,55,119,120]. After annealing above 1000 °C, crystallization of the amorphous Si inclusions occurs as evidenced by the narrowing and upshift of the Raman band to ~518 cm^–1^ (Figure 1). When measured with low laser intensity, the Raman band position (518–519 cm^–1^) is rather independent of the Si content and of the annealing temperature in the 1100–1200 °C range. According to the phonon confinement model [121,122], this result suggests that the Si-nc sizes evidenced by Raman spectroscopy do not change much under these conditions. However, growth of Si-nc with increasing annealing temperature was observed for SiO*_x_* films prepared by other deposition methods [37,40].

For MBD SiO*_x_* films with very small excess of Si (*x* > 1.9), the Raman bands of amorphous and crystalline Si are nearly invisible [116], in qualitative agreement with the earlier results [27]. The reason for this observation is the small amount of properly coordinated Si atoms (Si atoms bonded to four “bulk” Si atoms) for samples with low Si contents. A similar explanation was applied in a study of amorphous Si/SiO_2_ SLs, where the Raman bands of amorphous Si were observed for thicker Si layers (≥2 nm) and the Raman-scattering cross section decreased for thinner Si layers [55,123].

As the annealing temperature increases, the low-frequency shoulder in the Raman spectra responsible for disordered Si and/or small Si clusters gradually decreases (Figure 1). However, even after annealing at 1200 °C, the low-frequency component of the Raman band does not disappear and its area is comparable to that of the high-frequency component. As a possibility, small Si grains in a SiO_2_ matrix may be disordered even after annealing at this temperature whereas larger Si grains are crystallized.

**Figure 2:**
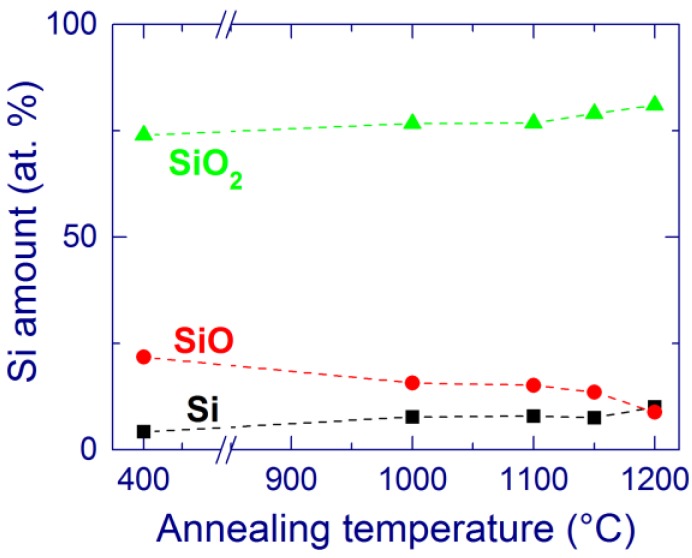
Composition of a SiO_1.7_ film provided by X-ray photoelectron spectroscopy (XPS) as a function of the annealing temperature. Reproduced with permission from [116]. Copyright 2012, AIP Publishing LLC.

X-ray photoelectron spectroscopy (XPS) confirms that the structural reorganization is not complete after furnace annealing (Figure 2). The Si–SiO_2_ phase separation increases with the annealing temperature, which is evidenced by the increasing amount of elemental Si and SiO_2_ and the decreasing amount of SiO. However, a large proportion of suboxides (10–15 at. % for *x* = 1.7) is still detected after annealing at 1200 °C [116]. This result indicates incomplete Si–SiO_2_ phase separation in these materials.

**Figure 3:**
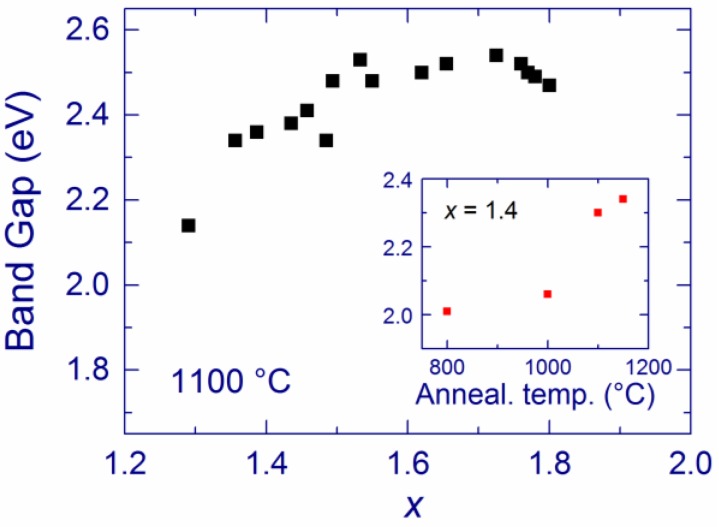
Band gap of SiO*_x_* films annealed at 1100 °C as a function of *x*. The inset shows the band gap of a SiO_1.4_ film as a function of the annealing temperature. Reproduced with permission from [116]. Copyright 2012, AIP Publishing LLC.

Additional information about the properties of SiO*_x_* films can be obtained from the band gap. For a given annealing temperature (from 900 to 1200 °C), the band gap of SiO*_x_* films increases as the Si content decreases [116]. For annealed at 1100 °C, the band gap increases from 2.2 to 2.6 eV as *x* increases from 1.3 to 1.8 (Figure 3), meaning that the Si-nc sizes somewhat decrease. According to the *ab initio* calculations for Si-nc with oxygen bonds at the Si/SiO_2_ interface, the obtained band gap change (2.2–2.6 eV) corresponds only to a marginal change of the Si-nc sizes, the average size being ~1 nm [124]. On the other hand, the position of the maximum of the Raman bands at 518–519 cm^–1^ corresponds to larger sizes (3–4 nm) [121,122], and this value is supported by TEM measurements of similar materials [125]. These different estimates can be connected with low sensitivity of the Raman and TEM methods to very small Si grains. The low-frequency Raman scattering at 490–500 cm^–1^ can be contributed by smaller Si-nc (1–2 nm) that may be partially disordered, which would agree with the Si-nc sizes derived from the band gaps. It should also be mentioned that the band gap of Si clusters is a complex function of a number of parameters in addition to the size, such as the crystallinity, strain due to the SiO_2_ matrix, and degree of oxidation [110,112]. Moreover, the Tauc law is generally applicable for amorphous semiconductors with absorption coefficients >10^4^ cm^–1^ [88]. Thus, the band gaps obtained by applying the Tauc law to crystalline Si grains with lower absorption may be somewhat inaccurate.

The band gap of SiO*_x_* (*x* ~ 1.4 [116] and *x ~* 1.8 [118]) films increases with the annealing temperature (see the inset in Figure 3). This increase can be explained by amorphous-to-crystalline transitions of Si clusters rather than by a decrease of their sizes, following the discussions by Mirabella *et al.* [33]. Indeed, the Si-nc sizes should rather increase with the annealing temperature [40,42] even though this increase for the MBD samples seems to be minor as suggested by the Raman spectra.

**Figure 4:**
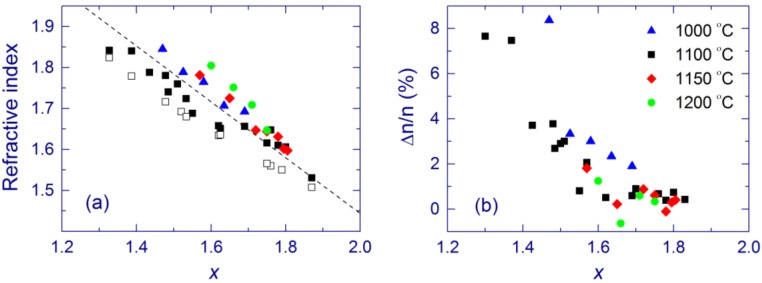
(**a**) TE refractive index and (**b**) birefringence of SiO*_x_* films annealed at 1000 °C (triangles), 1100 °C (squares), 1150 °C (diamonds), and 1200 °C (circles) as a function of *x*. The open squares show the refractive index obtained using the effective medium approximation and the XPS results for annealing at 1100 °C. The dashed line is a guide for the eye. (**a**) is reproduced with permission from [116]. Copyright 2012, AIP Publishing LLC.

By varying the Si content and annealing temperature, one can control the refractive index and the absorption coefficient of SiO*_x_* films. For a given annealing temperature, the refractive index increases with the Si content (Figure 4a) [118] as also reported previously [49,79]. Extrapolation of the data to *x* = 2 yields the refractive index of silica (1.456), which is a good verification of our results. The obtained values are also in agreement with other experimental results for annealing at 1100 °C, λ = 632 nm, and *x* ~ 1.38–1.63 [80]. For a given *x*, the refractive index typically decreases upon annealing at temperatures from 1000 to 1100 °C but increases in the 1100–1200 °C annealing temperature range [116].

The measured refractive index is interesting to compare with the values derived from the effective medium approximation and the chemical compositions measured by XPS (Figure 4a for 1100 °C and in Table 1 for *x* ~ 1.75). The agreement between the theory and the experiment is good. The observed small discrepancy can be due to the limitations of the used model (see Section 3.3). It is also possible that the refractive index of nanostructures is different from that of the bulk materials. Finally, in the XPS analysis, the mixture of different suboxides constitutes the SiO component, and this can also contribute to the error.

**Table 1:** Experimental refractive index *n*_exp_ and absorption coefficient α_exp_ of a SiO_1.75_ film for different annealing temperatures *T*_ann_ and the estimates for the refractive index *n*_est_ and extinction coefficient *k*_est_ from the effective medium approximation. Reproduced with permission from [116]. Copyright 2012, AIP Publishing LLC.

*T*_ann_ (°C)	*n*_exp_	α_exp_ (10^4^ cm^–^^1^)	*n*_est_	*k*_est_(SiO)	*k*_est_(Si)
400	-	1.57	1.575	0.45	1.25
900	-	0.56	1.57	0.07	1.25
1000	1.64 *	0.134 *	1.56 *	0	0.77
1100	1.61	0.126	1.56	0	0.53
1150	1.64	0.120	1.565	0	0.535
1200	1.67	0.128	1.57	0	0.48

* Obtained by extrapolation of the data to *x* = 1.75.

The SiO*_x_* film material prepared by MBD is found to be birefringent [79]. The classical theory of an isotropic asymmetrical planar waveguide predicts that the cut-off positions for the TE modes (with polarization parallel to the film) are at longer wavelengths than the TM cut-off positions (with polarization perpendicular to the film) [126,127]. However, the opposite order between the TE and TM peaks is found for some samples (see Figure 20a in Section 3.3). This reversed order of the TE and TM peaks is explained assuming positive optical birefringence, which is connected with the non-spherical shape of Si-nc [79]. Non spherical Si-nc have been observed in similar materials using the combination of electron tomography with plasmon-filtered microscopy [128]. It appears that that the optical birefringence is higher for SiO*_x_* films with the higher Si content and for lower annealing temperature (Figure 4b) [79,116]. Similar values of birefringence are obtained by *m*-line measurements [115].

The absorption coefficient of SiO*_x_* films can be analyzed using the effective medium approximation and the XPS data (Table 1). For an as-prepared SiO_1.75_ film, the extracted extinction coefficient of the SiO phase (0.45) is much larger than the value known for bulk SiO (0.055) if the extinction coefficient of amorphous Si (*k* = 1.25) is used for elemental Si. This mismatch would be smaller if the amount of elemental Si was underestimated by XPS and/or the extinction coefficient of nanoscale amorphous Si was larger than that of bulk amorphous Si. For annealing at 900 °C, our estimates agree with the extinction coefficients of bulk SiO and amorphous Si. For films annealed above 1000 °C, the SiO component is found to be very transparent, in contrast to bulk SiO, and this difference is possibly connected with ultra-small sizes of the suboxide regions.

As the annealing temperature increases, the estimated extinction coefficient of elemental Si decreases (Table 1), which is interpreted in terms of gradual crystallization of Si [115,116]. However, this process in not complete even after annealing at 1200 °C, because the calculated extinction coefficient (0.48 for *x* = 1.75) is much larger than that of crystalline Si (0.079); thus, Si is partially disordered, in agreement with the earlier results [42]. This “disordered” Si should differ from “ordinary” amorphous Si because no Raman band at 470 cm^–1^ is present for the films annealed above 1000 °C. Instead, the disordered Si grains may contribute to the Raman scattering at 490–500 cm^–1^, which is observed in Raman spectra of SiO*_x_* films annealed up to 1200 °C (Figure 1). As a possibility, ultra-small Si grains in a SiO_2_ matrix may be disordered even after the annealing whereas larger Si grains are crystallized. The presence of this disordered Si may explain the surprisingly large absorption of the annealed SiO*_x_* films. Moreover, the optical properties of bulk and nanoscale Si may differ, and the absorption by SiO inclusions cannot be completely excluded. This explanation of the relatively large absorption coefficient of annealed SiO*_x_* films is quite speculative, and additional experimental data are required to draw more accurate conclusions.

**Figure 5:**
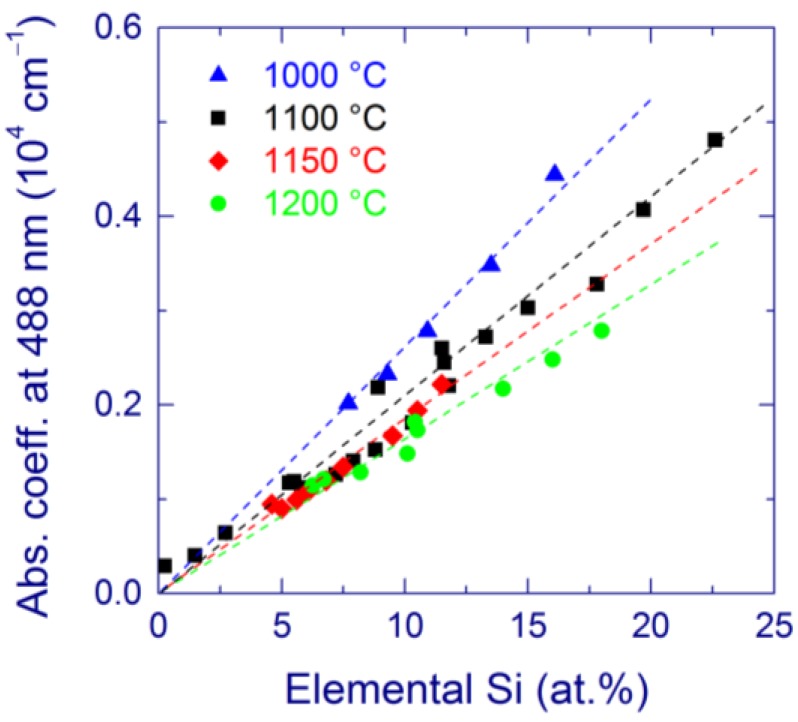
Absorption coefficient at 488 nm for annealing at 1000 °C (triangles), 1100 °C (squares), 1150 °C (diamonds), and 1200 °C (circles) as a function of the amount of elemental Si. The dashed line is a guide for the eye. Reproduced with permission from [116]. Copyright 2012, AIP Publishing LLC.

The absorption coefficient and the integrated Raman intensity of Si-nc are nearly proportional to the amount of elemental Si obtained by XPS (see Figure 5 for absorption) [115,116], in agreement with the earlier results [48]. It follows that elemental Si determines the absorption coefficient and the Si-nc Raman signal. A similar conclusion is made for SiO*_x_* films deposited by magnetron sputtering [129]. On the other hand, we have obtained that the Raman scattering cross-section of elemental Si in SiO*_x_* films prepared by MBD is about three times smaller compared to that of crystalline Si [116]. This difference can be explained by several factors. Ultra-small Si-nc (diameters < 2 nm) may be invisible by Raman spectroscopy due to the low proportion of “properly” coordinated Si atoms. The interaction with a SiO_2_ matrix can change the coordination of Si-nc, and this effect is stronger for smaller Si particles. In principle, the broad band gap of Si-nc may affect the Raman cross-section due to the resonance effect. Finally, the complex character of interaction of light with Si-nc embedded in SiO_2_ is not well understood. The present results are not enough to give a more reliable interpretation of these observations.

#### 2.1.2. Photoluminescence of the MBD Samples

**Figure 6:**
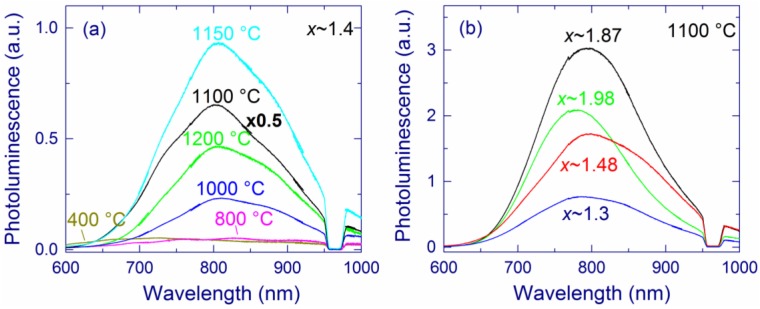
Photoluminescence (PL) spectra of (**a**) a SiO_1.4_ film annealed at different temperatures and (**b**) SiO*_x_* films with different *x* annealed at 1100 °C. The SiO*_x_* films are prepared by molecular beam deposition (MBD). The hole in the spectra around 970 nm is due to the second order of the notch filter. Notice the multiplication factor in (**a**). (**b**) is reproduced with permission from [116]. Copyright 2012, AIP Publishing LLC.

For SiO*_x_* films annealed up to 900 °C, weak PL with the maximum at 650–750 nm is observed (Figure 6a). This light emission is usually assigned to different thermodynamically unstable radiative defects, for instance, nonbridging oxygen-hole centers or E´-type defects [31,32,130,131,132]. After annealing above 900 °C, the PL maximum shifts towards longer wavelengths (~800 nm) and the PL intensity strongly increases [116,118], which agrees with numerous literature reports [27,34,42,53,72,91]. The highest PL intensity is observed for *x* ≅ 1.8–1.9 and annealing at 1100–1150 °C [116]. Annealing at 1200 °C decreases the PL intensity. A similar decrease of the PL intensity was also observed for SiO*_x_* (1.05 < *x* < 1.75) films prepared by magnetron sputtering [34]. A slightly different behavior is observed for SiO*_x_* (1.05 < *x* < 1.75) films prepared by PECVD. For these samples, the PL intensity increases with the annealing temperature up to 1250 °C [34,40].

**Figure 7:**
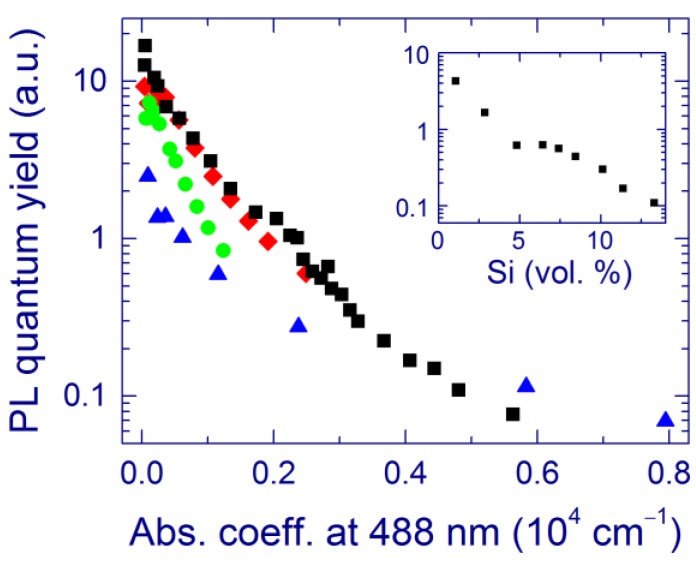
Relative PL quantum yield for SiO*_x_* films prepared by MBD for annealing at 1000 °C (triangles), 1100 °C (squares), 1150 °C (diamonds), and 1200 °C (circles) as a function of the absorption coefficient at 488 nm. The inset shows the relative PL quantum yield as a function of the amount of elemental Si for annealing at 1100 °C. Reproduced with permission from [116]. Copyright 2012, AIP Publishing LLC.

The enhancement of the 1.5-eV PL by annealing at ≥1000 °C is accompanied by the formation of Si-nc detectable by Raman spectroscopy [40,48,49,116,118]. This correlation seemingly suggests that the PL directly originates from these Si-nc. On the other hand, the PL intensity is the highest for samples with relatively small Si contents [48,49]. Moreover, for *x* > 1.9, no Si-nc and elemental Si are evidenced by the Raman and XPS measurements, respectively, whereas the PL is strong (Figure 6b). In fact, the PL quantum yield steadily increases as the absorption coefficient and the Si content decrease (Figure 7) [116,117]. These observations suggest that the Si-nc observed in the Raman spectra and detected by XPS as elemental Si are not direct light-emitting centers, which is in agreement with the defect origin of the 1.5-eV PL [52].

The PL position of our SiO*_x_* films is rather stable for different annealing temperatures (above 1000 °C) and Si contents (see Figure 6). These results seem to disagree with the studies of oxidized Si-nc prepared by magnetron sputtering [63]. Those experiments show that the PL position is size dependent for relatively large sizes (>3 nm) that are reliably measured by TEM. However, Wolkin *et al*. have shown that oxidation of Si-nc stabilizes the PL position for smaller Si-nc (<3 nm) [22].Thus, the stability of the PL position in our samples is consistent with the hypothesis that the observed PL originates from very small Si grains (<3 nm). In general, one has to distinguish different types of Si-nc samples. For example, the experiments on hydrogen-passivated Si-nc show that the PL of Si-nc with diameters smaller than 3 nm should be below 600 nm [23], which is not the case for our samples. This difference in PL positions can be explained by the decrease of the bandgap upon passivation of Si-nc with oxygen [110,133].

Now we discuss the possibility of assigning the 1.5-eV PL to defects and/or ultra-small Si grains. By analogy with oxidized porous Si [22,62], the light-emitting centers can be related to the Si=O covalent bonds [27,48] or to the bridge Si–O–Si bonds suggested by theory [106]. Ultra-small (below ~1 nm) oxidized Si grains considered by theory [114] is another candidate to explain the red PL. Several observations are consistent with the latter interpretation. First, the stable PL position for different Si content suggests small sizes of the light-emitting clusters (<3 nm) [22]. The calculated band gaps of Si clusters with diameters ~1 nm are 2–3 eV [115], which agrees with the band gaps obtained for our samples (Figure 3) [116]. The short PL lifetimes obtained for our samples (~1 μs) [117] also suggest that the PL originates from Si-nc that are quite smaller than 3 nm, for which the PL lifetime is ~40 μs [116,134]. Moreover, the theoretical study by Luppi *et al*. suggests that Si-nc with diameters up to 1 nm and with a Si–O–Si bridge bond at the surface emit light at ~1.5 eV [106], which is observed for our samples. The decrease of the PL intensity after annealing at 1200 °C is reasonable if the amount of small optically active Si-nc decreases at this temperature.

The direct excitation of the light-emitting centers by laser light is possible. As another excitation mechanism, laser light may be absorbed by relatively large Si clusters, and then the excitation is transferred to the light-emitting centers located at the Si-nc/SiO_2_ interface [68,135] or in more disordered areas [48,117]. Non-radiative defects such as dangling bonds at the Si‑nc/SiO_2_ interface as well as defects inside and outside the Si-nc can efficiently trap the excitation and therefore quench the PL [53]. Annealing at ~1100 °C presumably decreases the amount of these non-radiative defects and/or intensifies the energy transfer to the radiative centers, which enhances the PL. It has been shown that a considerable amount of non-paramagnetic near- and sub-gap defect states still remains after annealing, which drastically limits the PL quantum yield [136]. The amount of the light-emitting centers may increase upon annealing; however, no direct evidence of this is available.

Kusova *et al*. have suggested that compressive stress on Si-nc in a SiO_2_ matrix can redshift the PL [137]. However, it is difficult to estimate stress of very small Si-nc using Raman spectroscopy, due to their low Raman scattering cross-section. Such small Si-nc can contribute to the broad low-frequency shoulder at ~500 cm^–1^ (Figure 1). On the other hand, the Raman bands at 518–519 cm^–1^ observed in our samples feature unstressed Si-nc with sizes of ~4 nm [121] that are also detected by TEM [125]. It can be speculated that no substantial stress is exerted also on smaller Si-nc.

#### 2.1.3. Comparison of the Implanted and MBD SiO*_x_* Samples (*x* ~ 1.8)

**Figure 8:**
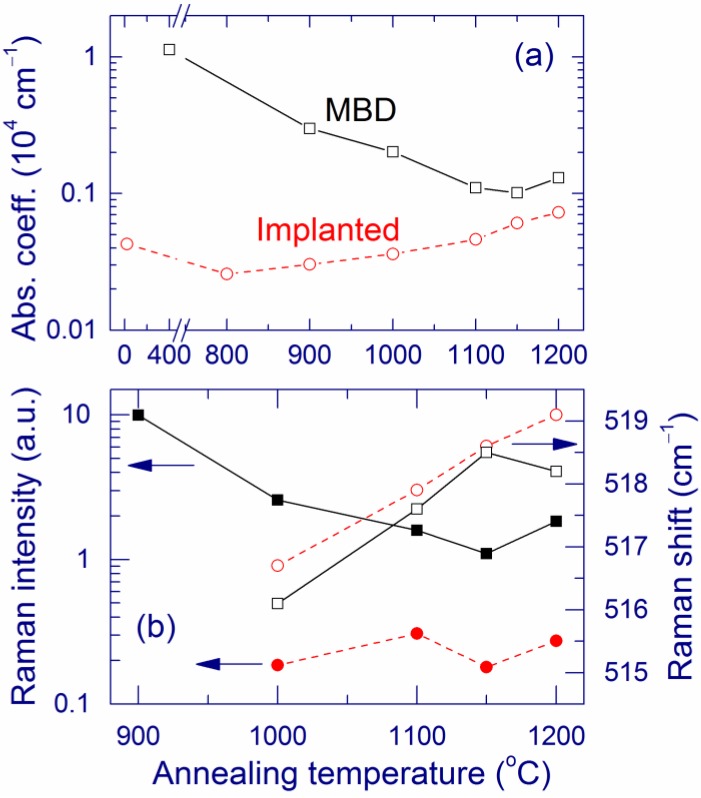
(**a**) Absorption coefficient at 488 nm for the MBD (squares) and implanted (circles) samples and (**b**) Normalized intensity (solid symbols) and position of the maximum (open symbols) of the Raman bands as a function of the annealing temperature. The Raman intensity is normalized by the effective film thickness (see Section 3.2). Reproduced with permission from [118]. Copyright Wiley-VCH Verlag GmbH & Co. KGaA.

After preparation, the structural and optical properties of the MBD and implanted samples are very different [118]. Amorphous Si clusters (>2 nm) are present in the as-grown MBD film, as indicated by a broad Raman band at ~470 cm^–1^ (Figure 1) and by a large absorption coefficient (Figure 8a). For the implanted samples annealed below 900 °C, the absence of Raman bands of Si and very low absorption (Figure 8a) suggest that only very small Si clusters (presumably with sizes up to ~1 nm) may be present in the material [55,123].

Upon annealing at 1000 °C, a narrow Raman band at ~516–517 cm^–1^ emerges for both types of samples, showing the formation of Si-nc. As compared to the as-prepared samples, the absorption coefficient of the annealed samples is increased for ion implantation but it is decreased for MBD (Figure 8a). After annealing at 1100–1200 °C, both types of samples become optically and structurally more similar. For both types of samples, the Raman band is observed at ~518 cm^–1^ and the absorption coefficients become closer (Figure 8). These trends are reasonably explained by growth and crystallization of Si clusters in the implanted sample and by crystallization of amorphous clusters in the MBD sample. Indeed, the extinction coefficient of crystalline Si is much smaller than that of amorphous Si.

After annealing (>1000 °C), the normalized Raman intensity and the absorption coefficient are larger for the MBD samples compared to the ion-implanted sample (Figure 8). This result indicates that the amount of Si-nc with diameters of ≥2 nm is greater in the MBD films. This conclusion is also supported by a smaller band gap for the MBD sample (~2.6 eV) compared to that for the implanted sample (~3.3 eV). For both types of samples, a substantial amount of suboxides (~15%) is detected by XPS even after annealing at 1200 °C, indicating that the Si–SiO_2_ phase separation is not complete.

**Figure 9:**
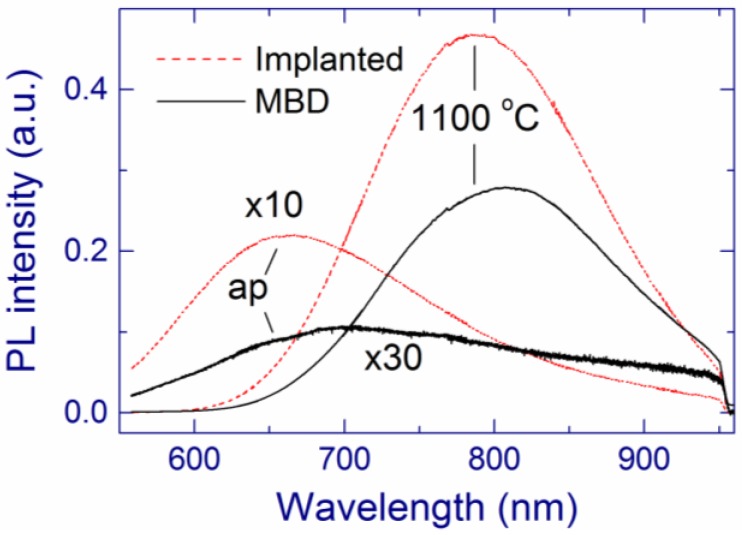
PL spectra of as-prepared (ap) and annealed (1100 °C) SiO_1.8_ films fabricated by MBD and ion implantation. The PL spectra are normalized by the effective film thickness (see Section 3.2). Reproduced with permission from [118]. Copyright Wiley-VCH Verlag GmbH & Co. KGaA.

For annealing below 900 °C, the PL maximum is located at about 650–700 nm for both types of samples, and the PL intensity is much higher for the implanted sample (Figure 9). The latter fact indicates a larger amount of thermodynamically unstable radiative defects in the implanted sample [31,32,130,131,132]. Annealing at ~1100 °C enhances the 1.5-eV PL for both types of samples. After this annealing, the normalized PL is slightly stronger for the implanted sample (Figure 9), which is remarkable because the Raman band of Si-nc is weaker for this preparation method (Figure 8b). Annealing at 1200 °C weakens the PL, and this effect is more pronounced for the implanted sample. This decrease of the PL can originate from a thermal modification of the light-emitting centers, for example, an increase of the size of oxidized ultra-small Si grains. In the case of the implanted samples, this conclusion is supported by the redshift of the PL to ~900 nm and by the decrease of the band gap from 3.3 to 2.8 eV for annealing at 1150 and 1200 °C, respectively.

#### 2.1.4. Laser Heating of Films on Substrates

**Figure 10:**
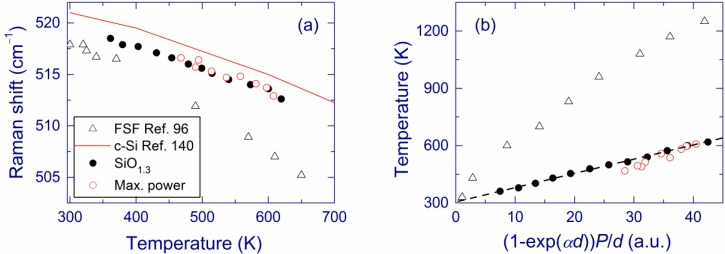
(**a**) Raman shift as a function of the laser-induced temperature and (**b**) Laser-induced temperature as a function of the absorbed laser power. The measurements are made for a SiO_1.3_ film on a silica substrate annealed at 1100 °C using various 488-nm laser intensities (solid circles) and for SiO*_x_* films (1.3 < *x* < 1.5) at the maximum laser intensity of ~10^4^ W cm^–2^ (open circles) [116]. The data for a free-standing SiO_1.7_ film [95] (FSF, triangles) and for crystalline Si [139] (solid line) are shown for comparison. The dashed line in panel b is a linear fit. Reproduced with permission from [116]. Copyright 2012, AIP Publishing LLC.

Laser light is found to increase the temperature of SiO*_x_* films on silica substrates [116]. For example, 488-nm laser power of ~130 mW focused to a ~40-μm spot (laser intensity ~10^4^ W cm^–2^) leads to a temperature of ~350 °C in a SiO_1.3_ film annealed at 1100 °C. The absorption coefficient of this film increases at 350 °C by ~40% compared to the room temperature value, which is probably due to the thermally induced shrinking of the bang gap [97]. At this laser-induced temperature, the Raman band of Si-nc is observed at ~512.5 cm^–1^, *i.e*., it is down-shifted by ~6 cm^–1^ from the room temperature position. The temperature dependence of the Raman shift (Figure 10a) is in excellent agreement with the calculations by Faraci *et al*. for Si-nc with sizes of 5 nm [138]. Similar temperature dependences were previously reported for crystalline Si [139] and the free-standing SiO_1.7_ film [95]. The phonon confinement effect and residual stresses can explain the quantitative differences between the Raman band positions in these cases.

Figure 10b shows the laser-induced temperature for a free-standing SiO1.7 film and SiO*x* films (1.3 ≤ *x* < 1.5) on silica substrates as a function of the laser power absorbed by a unit volume, which is proportional to $\frac{(1-e^{\alpha d})P}{d}$, where *P* is the laser power, α is the absorption coefficient, and *d* is the film thickness [95]. The linear fit of the temperature *versus* the absorbed power yields the room temperature at zero power, which verifies the results. For the same absorbed power, the laser-induced temperature is much higher in the free-standing films compared to the films on substrates, which is due to the thermal flow to the substrate in the latter case.

In Raman measurements of Si-nc, relatively high laser powers are usually employed to obtain a good signal-to-noise ratio. It follows that the laser-induced heat should always be carefully considered even for films on substrates (especially when using Raman microscopes) to obtain reliable information of the material structure. In general, laser heat, local stress, and phonon confinement are overlapping effects with respect to the Raman spectra.

### 2.2. Laser Annealing of Free-Standing Films

#### 2.2.1. Laser-Annealed Areas

**Figure 11:**
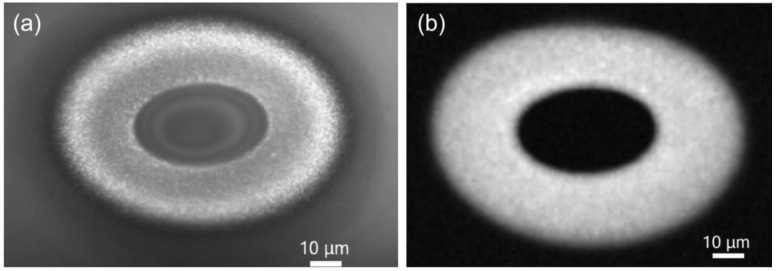
(**a**) Optical microscope photograph and (**b**) Raman map of the Si-nc band. A 2-μm-thick free-standing SiO*_x_* film (*x* ~ 1.7–1.8) is exposed to 488-nm light (~110 mW) through a lens (focal length of 150 mm). In the optical photograph, the sample is illuminated through the objective. The oval shape is due to a non-perpendicular direction of the annealing laser beam. Brighter regions in the Raman map correspond to stronger Raman signals. The Raman signal is integrated in the 510–540 cm^–1^ spectral range. Reproduced with permission from [140]. Copyright 2010, AIP Publishing LLC.

We have studied CW laser annealing (488 nm) of free-standing SiO*_x_* films (0.5 and 2 μm-thick) and Si/SiO_2_ SL (0.5-μm-thick). After preparation (Section 3.1), the free-standing films presumably contain small Si-nc (up to 4 nm in diameter) [95,125]. The laser annealing is usually performed for ~1 s in air atmosphere (if different is not stated). An optical photograph of a typical laser-annealed area on a free-standing film and a Raman map from the same area are presented in Figure 11. Three regions can be distinguished: (i) central region, (ii) ring around the central region, and (iii) pristine film outside the irradiated area [140,141,142].

**Figure 12:**
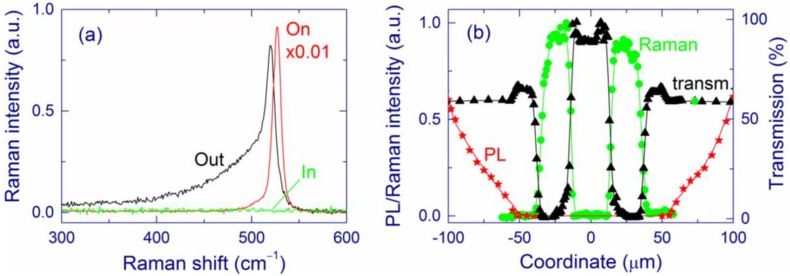
(**a**) Raman spectra of a laser-annealed 2-μm-thick SiO*_x_* (*x* ~ 1.7–1.8) film from regions in the center (in), on the ring (on), and outside the laser-annealed area (out). (**b**) Raman (circles), PL (stars), and transmission (triangles) cross-sections of the laser-annealed area. The cross-sections are measured in the vertical direction through the center of the laser-annealed area shown in Figure 11. Reproduced with permission from [140]. Copyright 2010, AIP Publishing LLC.

In the central region, the material is essentially amorphous SiO_2_. For 0.5-μm-thick SiO*_x_* films (*x* ~ 1.7–1.8), this composition is directly shown by XPS and electron energy loss spectroscopy (EELS) [140]. In accord, the TEM images and selected area diffraction (SAD) patterns indicate a very homogeneous and amorphous structure of this region, and the Fourier-transform infrared spectra (FTIR) measured with high spatial resolution are similar to those of thermal silica. In this region, no Si-related peak is detected by Raman spectroscopy, the absorption is very low, and no PL is observed (Figure 12). In agreement, for 0.5-μm-thick Si/SiO_2_ SL (2-nm-thick Si and SiO_2_ layers), no Si clusters are detected in bright-field scanning TEM (BF-STEM) images, and energy-dispersive X-ray spectroscopy (EDS) indicates an increase of *x* in the central region compared to the pristine film [141,142]. Quantitative analysis based on the EDS measurements is not possible because quantification using low-energy X-ray lines such as oxygen K alpha is not feasible. Small Si clusters (<1 nm) invisible by these microscopic methods are in principle possible in the central region. However, very small Si clusters are thermodynamically unfavorable [143], and their formation seems to be improbable at high temperatures. Furthermore, these very small Si grains are presumably luminescent; however, practically no PL is detected from the central region.

**Figure 13:**
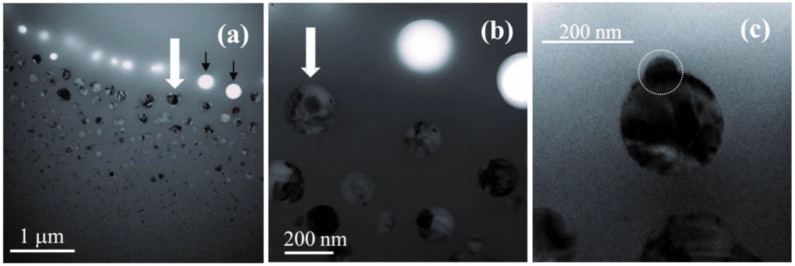
TEM images of a 0.5-μm-thick Si/SiO_2_ SL showing a Si particle located near the film surface (marked with a white arrow). The images in (**a**), (**b**), and (**c**) are taken with different magnification. (**c**) shows this particle in the tilt geometry, the surface subparticle being indicated by a dotted circle. The bright spots are holes in the film; the holes observed in (**b**) are marked by black arrows in (**a**). Figure courtesy of Simona Boninelli and Fabio Iacona. Reproduced with permission from [141]. Copyright 2012, AIP Publishing LLC.

In the ring around the central region, the EFTEM, BF-STEM, and TEM images evidence large Si-nc embedded in amorphous SiO_2_ (Figure 13) [140,141,142]. The maximum Si-nc diameters (~200 nm) for the 0.5-μm-thick Si/SiO_2_ SLs [141] are on average two times larger than those detected for the 0.5-μm-thick free-standing SiO*_x_* films (*x* ~ 1.7–1.8) [140]. This difference is reasonably explained by a larger Si content in Si/SiO_2_ SLs (*x* ~ 0.7 from the deposition conditions) compared to the SiO*_x_* films (*x* ~ 1.7–1.8). The crystallinity of the large Si clusters in the ring region is confirmed by selected-area diffraction measurements (for 0.5-μm-thick SiO*_x_* film) and Raman spectroscopy. The Raman band is quite narrow and does not have a shoulder at 450–500 cm^–1^, indicating the absence of amorphous phase and therefore suggesting efficient Si crystallization (see Figure 12a). The EELS spectra show the presence of the Si and SiO_2_ phases. In accord, the FTIR spectra correspond to stoichiometric SiO_2_ perturbed by the Si–SiO_2_ interface. The Raman bands from the ring region is stronger by about 2–3 orders of magnitude compared to the pristine film, which is explained by the resonance size effect for the large Si-nc [144]. The Raman bands from Si-nc in the ring region are typically shifted up to ~530 cm^–1^ (from the value of 521 cm^–1^ for crystalline Si) indicating a compressive stress on Si-nc. According to the mechanism proposed earlier [56], these large Si-nc are formed from the liquid phase, and the stress appears when their volume suddenly increased in the solid SiO_2_ matrix. Thus, the temperature in the ring region exceeds the melting point of Si (1685 K). Indeed, a temperature of ~2000 K was estimated by fitting the strong light-emission background upon irradiation with high laser intensities (~16 kW cm^–2^ at the sample) [94]. The bright thermal radiation is also seen by the naked eye. High processing temperatures in the central region are also confirmed by the decrease of the film roughness detected by AFM. For 0.5-μm-thick Si/SiO_2_ SLs, scanning electron microscopy (SEM), TEM, and atomic force microscopy (AFM) show several large holes (up to 200 nm) in the inner part of the ring region (Figure 13a,b).

These results demonstrate a Si–SiO_2_ phase separation on a macroscopic scale. The central region (up to 30 μm) is cleaned of the Si excess. On the other hand, in the ring region, large Si-nc are formed and the Si excess is enhanced as found for 0.5-μm-thick SiO*_x_* film by XPS and EELS [140]. The macroscopic phase separation can be explained as follows [140]. In the beginning of laser irradiation, the film structure is homogeneous so that the temperature gradient approximately corresponds to the intensity gradient in the Gaussian beam. The temperature gradient gives rise to a concentration gradient due to different diffusion properties of the mixture constituents. For example, Si, O, and SiO can diffuse in silica [145], which changes the relative concentrations. Diffusion in temperature gradient is known as thermodiffusion or Ludwig-Soret effect [146,147]. As detected by EELS, XPS, and AFM, the central region is typically thinner than the pristine film by about 30%–40% and ~50% for 0.5-μm-thick Si/SiO_2_ SLs and SiO*_x_* films, respectively. Moreover, AFM shows an increase of the film thickness in the ring region compared to the pristine film [142]. These results are also indicative of macroscopic movement of Si excess from the central region.

#### 2.2.2. Surface Structure

For laser-annealed 0.5-μm-thick Si/SiO_2_ SLs, surface analysis (SEM and AFM) has been used to discriminate Si-nc located near the film surface [141]. In the tilt TEM images (Figure 13c), these “surface Si-nc” have an unusual pear-like shape with the thinner part presumably sticking out of the laser-illuminated surface. The AFM and SEM methods estimate that the thinner part above the surface (surface feature) has a typical size of 60–70 nm, in agreement with the TEM measurements. The subsurface part has a diameter of ~200 nm, as shown by TEM and BF-STEM images.

The formation of the surface features is explained by the following mechanism. Since most of large Si-nc formed by laser annealing are under high compressive stress, it is possible that the laser-pressurized silicon can erupt from the film if it is separated from the ambient atmosphere by a very thin silica layer. The erupted Si is seen as the subparticle in Figure 13c. The volume of the subparticle (~70 nm in diameter) is ~5% of the volume of the larger subsurface part of the nanocrystal (~200 nm in diameter). Eruption of this volume would lead to a substantial decrease of the compressive stress of the parent particle [56]. The fact that no eruptions are detected on the backside of the film seems to be consistent with this mechanism. Indeed, the temperature should be somewhat higher on the front (laser-illuminated) side of the film; hence, the covering silica film is softer here, which favours Si eruption.

**Figure 14:**
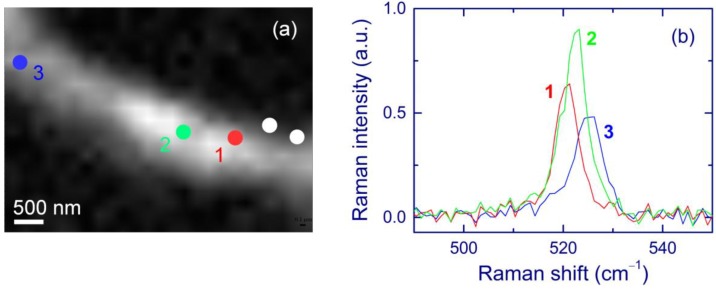
Raman characterization of the area shown in Figure 13a. (**a**) Raman map of Si-nc (integration from 515 to 530 cm^–1^) obtained with a 100× objective and steps of 0.2 μm. Brighter areas correspond to stronger Raman signals. The white circles indicate the positions of two holes used as the reference points. (**b**) Raman spectra in positions 1, 2, and 3 indicated in (**a**). Position 1 corresponds to the surface Si-nc shown in Figure 13. Reproduced with permission from [141]. Copyright 2012, AIP Publishing LLC.

Raman measurements confirm the proposed eruption model. The surface Si nanocrystal shown in the TEM images in Figure 13 is located in position 1 in Figure 14a. The Raman shift obtained in this position is ~520 cm^–1^ (spectrum 1, Figure 14b), which indicates a low stress. It is reasonable that Si eruption can relax stress in the vicinity of surface Si-nc. The compressive stress is partially relaxed at a distance of about 1 µm from the surface feature (spectrum 2). The Raman spectrum measured at position 3, which is far away from the surface feature, shows a higher compressive stress (spectrum 3 in Figure 14b).

#### 2.2.3. Effect of the Annealing Atmosphere

The studies described above have been performed in the atmosphere of air (containing ~20% of oxygen), which can cause some oxidation of Si during laser annealing. Indeed, oxygen can diffuse through the film oxide to Si-nc [148,149,150]. This process may decrease the excess of Si, especially in the central region, where the temperature in the beginning of laser annealing is the highest. To evaluate this effect, we compare laser annealing in atmospheres containing different amounts of oxygen (Ar, N_2_, O_2_, and air). Detailed measurements are performed for 0.5-μm-thick Si/SiO_2_ SL and 1-s exposure [142].

For all annealing atmospheres, the central region of laser-annealed areas most probably consists of amorphous silica without Si excess. This composition is indicated by the absence of the Raman signal of Si in this region, an increase of *x* compared to the pristine film detected by EDS, and the absence of Si clusters in the BF-STEM images. In the ring region, large Si-nc (up to 150–300 nm) are formed, and the film thickness tends to increase compared to that of the pristine film. The change of the film thickness was estimated using the AFM images taken from both sides of the film. These results support the concept of macroscopic Si–SiO_2_ phase separation as a result of laser annealing.

The effect of oxygen can be analyzed by comparing the film thickness in the central region obtained for different atmospheres. Indeed, two competitive processes, thermodiffusion and Si oxidation, affect the film thickness in the opposite directions, tending, respectively, to decrease and to increase it. For Ar atmospheres, the central region is thinner by ~150 nm than the pristine film, *i.e*., the thickness is about 350 nm. This observation supports the concept of the macroscopic phase separation because Si oxidation does not operate in the inert atmospheres. A similar change of the thickness occurs in air (the thickness of the central region is 300–350 nm) also indicating minor Si oxidation.

In contrast, laser annealing in pure O_2_ increases the film thickness in the central region by ~20 nm compared to the pristine film. Thus, both thermodiffusion and Si oxidation seem to operate in this case, practically compensating each other. It seems that a significant part of Si is oxidized upon laser annealing in pure oxygen. The difference between the film thickness of the central region for inert and O_2_ atmospheres is ~200 nm (larger in oxygen). 200 nm of SiO_2_ can be formed by oxidation of about 100-nm Si layer, which corresponds to ~40% of all amount of elemental Si in the pristine SL (125 2-nm-thick Si layer). According to the model suggested by Deal and Grove for crystalline Si, 1 bar of dry O_2_ at 1200 °C produces ~20-nm oxide film during 1 s, whereas a ~33-nm oxide is formed for 100 s [149], which is in approximate agreement with our estimates. It should be reminded that the temperature of laser annealing is sufficiently higher [94]. In addition, the extent of oxidation can differ from these values because our pristine material is very different from crystalline Si.

The structure of the ring region is also affected by the amount of O_2_ in the ambient atmosphere. For inert atmospheres, the AFM and BF-STEM images show that the ring region is very inhomogeneous. It has areas with large Si-nc (up to 150 nm in diameter), with large non-spherical holes up to 300 nm, and with smaller Si-nc (up to 50 nm in diameter). No surface Si-nc are observed in this case, as shown by AFM and SEM. Raman spectroscopy indicates Si-nc with different stresses (Raman shifts from 518 to 530 cm^–1^) and regions with very different intensities of Raman bands of Si-nc. The BF-STEM images show a “belt” of nano-channels (or pores) around the ring region. The size of these nano-channels can be as low as several nanometers.

**Figure 15:**
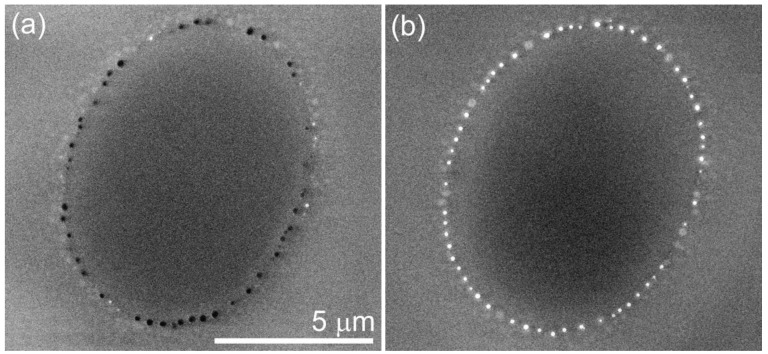
Secondary electron (SE) images of the front side of laser-annealed areas produced in (**a**) air and (**b**) O_2_ atmospheres during 1-s exposure. Surface features are white, holes are black. The scale is the same in both panels. Figure courtesy of Marianna Kemell. Reprinted with permission from [142]. Copyright 2014, American Scientific Publishers.

For laser annealing in air, the ring region contains Si-nc with diameters of up to 200 μm. A chain of holes with sizes up to 200 μm is seen at the inner part of the ring of large Si-nc (Figure 15a). A number of surface features are observed as discussed above.

For pure O_2_ atmosphere, in addition to Si-nc inside the film, SEM and AFM show a regular chain of surface features that are located in the inner part of the ring at the front side of the film (Figure 15b). The undersurface part of the surface Si-nc can be up to 300 nm in diameter. In agreement with the suggested eruption model, Raman spectroscopy shows a relaxed stress for these surface Si-nc (Raman shift of ~520 cm^–1^) whereas the Si-nc at the outer part are still under compressive stress (Raman shift up to ~530 cm^–1^). No holes in the film are observed after laser annealing in O_2_ atmosphere.

Based on these results, it can be suggested that the holes produced in inert and air atmospheres are formed via removal of surface Si-nc from the film. For laser annealing in air, the holes appear in the positions where a chain of large surface Si-nc is observed in the case of O_2_ atmosphere (see Figure 15). The laser-annealed areas with larger number of holes contain less surface Si-nc, and the presence of oxygen increases the amount of surface Si-nc. This observation suggests that oxygen decreases the removal of large Si-nc by the formation of a thin oxide layer covering surface Si-nc, which “glues” them to the film. The large irregular holes obtained in the inert atmospheres also presumably originate from large Si-nc removed from the film. The suggested mechanism of hole formation is consistent with the dependence of the maximum Si-nc sizes on the annealing atmosphere. For inert atmospheres, the largest Si-nc visible in the BF-STEM images are “only” ~150 nm in diameter because bigger Si-nc are removed from the films forming holes with sizes of up to 300 nm. For O_2_ atmosphere, the largest Si-nc (up to 300 nm) are detected because they are protected by thin oxide layers. Annealing in air represents an intermediate case where the largest Si-nc are ~200 nm in diameter, and some Si-nc are removed forming holes. In accord, with the proposed mechanism, holes are not observed in a 0.5-μm-thick SiO*_x_* film (*x* ~ 1.7–1.8) annealed in air [140]. For this film, Si-nc have diameters “only” up to 100 nm, which are substantially smaller than the film thickness. These Si-nc are mostly located below the film surface and, hence cannot be removed to form holes.

**Figure 16:**
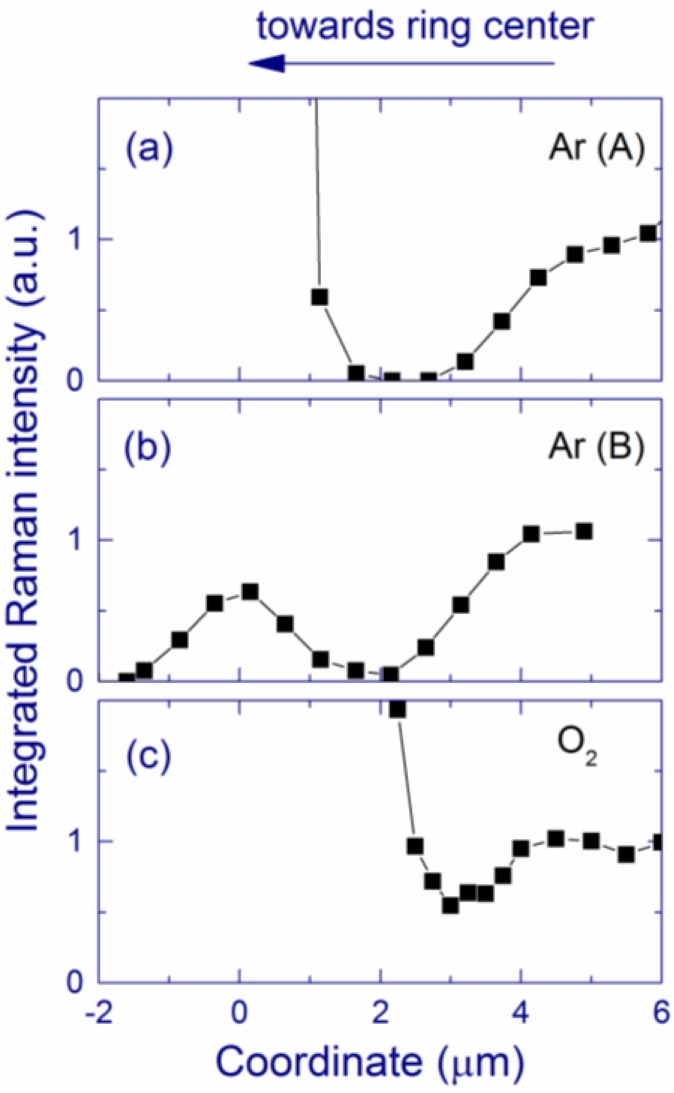
Characteristic Raman cross-sections of the ring region. Two characteristic cross-sections (**a**) and (**b**) are shown for Ar atmosphere, and the cross-sections (**c**) are similar for O_2_. For air, the cross-sections are similar to the case of O_2_. The Raman signal is integrated in the 230–585 cm^–1^ range. The data are normalized by the Raman intensity measured from the pristine film. Reprinted with permission from [142]. Copyright 2014, American Scientific Publishers.

Figure 16 shows the cross-sections of the Raman signal measured through characteristic ring regions of the laser-annealed areas prepared in inert (Ar) and O_2_ atmospheres. For inhomogeneous ring regions formed in Ar atmosphere, two different characteristic cross-sections are observed (Figure 16a,b) whereas for O_2_ and air atmospheres, the laser-annealed area is more uniform having similar Raman cross-sections in different ring regions (see Figure 16c for O_2_). For 488-nm laser light, the Raman enhancement is known to be the strongest for spherical Si particles with diameters of ~112.5 nm [144]. In agreement, Si-nc with sizes ~100 nm are observed in the areas with the strongest Raman signal (at coordinate = 0). At coordinates >5 μm, the Raman spectra are similar to that of the pristine film. In Figure 16b, the Raman intensity is relatively weak in the ring region because the Si-nc diameters in this case do not exceed ~50 nm, which is smaller than the resonance value of ~112.5 nm.

For inert atmospheres (Figure 16a,b), the Raman signal is very weak at coordinates 2–3 μm. For air and O_2_, this decrease of Raman intensity is less pronounced but still observable (Figure 16c). We call this effect the secondary Si depletion, and it supports the model of macroscopic phase separation. We tentatively explain the formation of this interval with low Raman intensity in the following way. When the large Si-nc are formed in the ring region during laser annealing, the absorption coefficient in this area strongly increases resulting in an increase of the temperature. Due to the thermal conductivity, the temperature of the vicinity also increases, which leads to a temperature gradient and additional macroscopic phase separation outside the ring region. This process seems to be more efficient for the inert compared to oxygen-containing atmospheres. The position of this spatial interval roughly coincides with the position of the belt of nano-pores and nano-channels observed by SEM in the inert atmospheres. The exact mechanism of the formation of these pores is unclear.

#### 2.2.4. Effect of the Exposure Period

Laser annealing with different exposures (10 ms and 100 s, in addition to 1 s) has been studied for 0.5‑μm-thick Si/SiO_2_ SL mostly in air atmosphere [142]. For the central area, the results are similar for all exposures: the EDS measurements show an increase of *x* (relative to the pristine film), no Raman signal from Si-nc is detected, and no Si clusters are visible in the BF-STEM images. Thus, 10-ms exposure is enough for the removal of excess Si from the central region. In other words, the formation of the central region occurs in a millisecond time scale.

On the other hand, the ring region differs for different exposures. For 10-ms exposures, no holes and surface features are found and Si-nc have diameters of up to 20–30 nm. For 1-s and 100-s laser exposures, some holes and surface features are formed and Si-nc with diameters up to 200 and 250 nm, respectively, are observed. Thus, longer laser exposure increases the Si-nc size. Coalescence of Si-nc can be suggested for this process [73], because the number of Si-nc decreases for longer exposure periods. The increase of the Si-nc sizes with the exposure period can probably explain the greater amount of surface Si-nc observed for 100-s compared to 1-s exposure. For other atmospheres, the prolonged laser annealing (100 s) also decreases the amount of small Si-nc and increases the average sizes of Si-nc compared to 1-s exposure.

**Figure 17:**
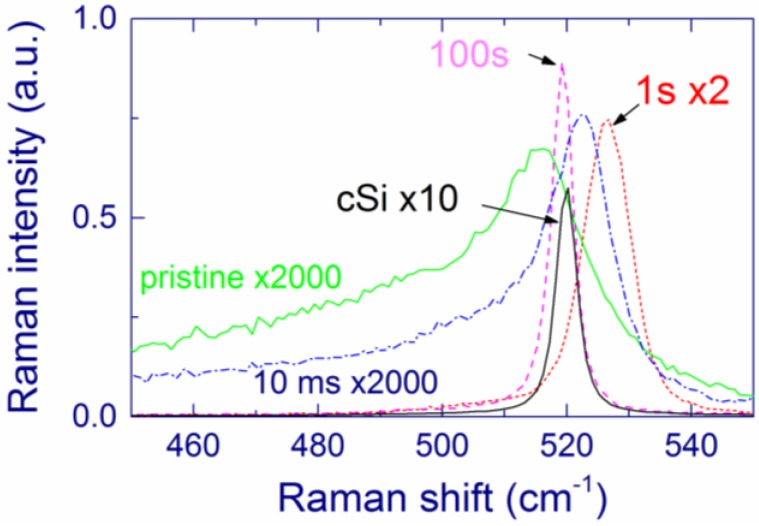
Typical Raman spectra of the ring region for 10-ms, 1-s, and 100-s exposures in air. Raman spectra of the pristine film and crystalline Si wafer (cSi) are also shown. Reprinted with permission from [142]. Copyright 2014, American Scientific Publishers.

The Si-nc diameters for 10-ms exposure (20–30 nm) are smaller than the resonance size of 112.5 nm. This explains why no Raman enhancement is observed from these Si-nc (Figure 17). In contrast, for 1-s and 100-s exposures, the Raman intensity is strongly enhanced compared to that from the pristine film, which is explained by the presence of Si-nc with diameters of ~100 nm. The Raman band observed after 10-ms exposure has a smaller low-frequency shoulder compared to the pristine film, meaning that the amount of disordered Si and/or small Si clusters is reduced. As the exposure increases, the Raman band narrows indicating that the structure of Si-nc becomes more regular. For 100-s exposure, the width of the Si-nc Raman band is as narrow as that of a crystalline Si wafer, which indicates an almost perfect crystalline structure.

For 10-ms and 1-s exposures, the Si-nc in the ring region are under compressive stress because the Raman shifts are larger than that of crystalline Si (Figure 17); thus, these Si-nc are formed from the liquid phase. On the contrary, no stress on Si-nc is detected for 100-s exposure for all studied annealing atmospheres. This fact may show that for the long exposure, the temperature of large Si-nc becomes at some moment below the melting point. The decrease of the temperature for long exposure may be explained by the decrease of the absorption when the Si-nc become much larger than the resonance size of 112.5 nm [144]. Annealing at temperatures below the melting point relaxes the stress [56,96].

#### 2.2.5. Optical Memory

**Figure 18:**
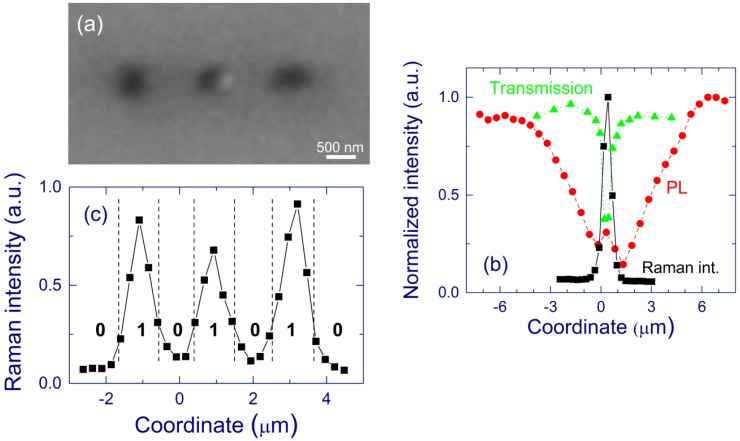
Writing digital data on a free-standing 2-μm-thick Si/SiO_2_ SL. (**a**) Camera photograph of three spots produced by 488-nm laser annealing for ~10 ms. (**b**) Cross-section of the Raman, transmission, and 1.5-eV PL signals through a typical laser spot. (**c**) Cross-section of the Raman intensity through the spots presented in panel a, which illustrates digital data points. The Raman intensity is integrated in the 500–540 cm^−1^ range. Reproduced with permission from [151]. Copyright 2009, AIP Publishing LLC.

We describe two concepts of optical memory based on laser annealing of Si nanostructures in free-standing Si/SiO_2_ SLs [151]. The first concept is based on the enhancement of Raman signal. The sample is irradiated by a 488 nm laser beam focused by a 100× objective of a Raman microscope, which produces laser-annealed areas with sizes below 1 μm. These areas are clearly visible in camera photographs (Figure 18a), and this indicates a change in the reflection and/or absorption coefficient of the film. The Raman, PL, and transmission profiles through a typical laser-annealed spot are presented in Figure 18b.The Raman signal is strongly enhanced whereas the transmission is decreased due to temperature-induced Si–SiO_2_ phase separation and ordering and growth of Si-nc [140,141,142].

The observed laser effect is a case of non-volatile memory. Binary digits can take values of either 0 or 1 as demonstrated in Figure 18c. The smaller Raman intensity corresponds to 0, whereas the higher Raman intensity represents 1. Similar data reading can be obtained by transmission or reflection measurements. The estimated data density for this method is ~1 bit/μm^2^, which can be improved by using stronger objectives and shorter laser wavelength. The nature of the used material provides high thermal stability of the described optical memory. The thermal stability of the laser-crystallized spots was tested by furnace annealing for 1 h at 1200 °C in a nitrogen atmosphere. As expected, Raman and transmission signals remained unchanged. This approach does not allow erasing and rewriting information.

**Figure 19:**
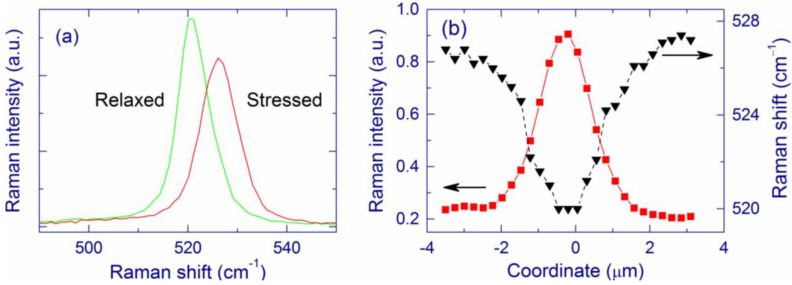
(**a**) Raman spectra of Si-nc with stress of ~3 GPa and after laser-induced relaxation (for 2-μm-thick Si/SiO_2_ SL). (**b**) Cross-sections of the Raman band position and Raman intensity integrated in the 510–520 cm^−1^ region. Reproduced with permission from [151]. Copyright 2009, AIP Publishing LLC.

The second concept is based on Si-nc stress. First, a free-standing Si/SiO_2_ SL is shortly irradiated with a relatively large laser spot (10–40 μm in diameter), which produces an extended area with a compressive stress of ~3 GPa (see Figure 11). This stress is evidenced by Raman bands at ~527 cm^−1^ (Figure 19a). Next, the stressed area is irradiated by a highly focused laser beam using a 100× objective and reduced laser power. As a result of laser annealing below the Si melting temperature, the stress is released in the irradiated spot and the Raman band shifts down to ~518 cm^–1^. The typical size of the spot with relaxed stress is ~1 μm (Figure 19b). The optical constants are practically unchanged upon the stress relaxation, and this effect is invisible in optical photographs. The laser-induced decrease of the Raman shift writes data pixels on the film similarly to the concept based on the Raman enhancement. The data can be erased by inducing stress using a broad laser beam and then re-written again. The retention time of the stress-based memory is estimated to be about 1 year at ~300 °C [56].

## 3. Experimental Section

### 3.1. Samples

Most of the samples used in this study have been prepared by MBD in the Aalto University (Finland) by S. Novikov. The SiO*_x_* films are deposited on silica wafers. Some of the samples have a gradient of the Si content in the lateral direction, and they are called optical wedges. Due to the deposition conditions, these films also have a varying thickness (1.5–2.5 μm). The advantage of the optical wedges is that the areas with different Si contents are annealed under the same conditions and the O/Si concentration ratio *x* can be continuously changed by selecting the film area. The wedged samples are cut in slices (5–7 cm long and ~1 cm wide) along the Si content gradient and annealed in a furnace at temperatures up to 1200 °C for 1 h in nitrogen atmosphere. In [116], three samples covering *x* from ~1.3 to ~1.98 are studied.

The free-standing films are prepared from the SiO*_x_* films and Si/SiO_2_ SL sdeposited on Si wafers and annealed in a furnace at 1100 °C in nitrogen atmosphere. The free-standing films are made by chemical etching of the Si substrate [125,140,141,142]. The Si/SiO_2_ SLs are constituted of 125 or 500 pairs of 2-nm-thick Si and SiO_2_ layers, yielding the total film thicknesses 0.5 or 2 μm, respectively. The thicknesses of the free-standing SiO*_x_* films (*x* ~ 1.7–1.8) are 0.5 and 2 μm.

One sample is made by Si-ion implantation into a silica substrate in the Accelerator Laboratory (University of Helsinki) [118]. The implantation of Si ions into a silica plate was done with different energies (30, 56, 98, 160, 250, and 400 keV) and doses to achieve a nearly constant Si excess in the SiO*_x_* layer with a thickness of ~750 nm. The O/Si concentration ratio *x* estimated by XPS is ~1.8. The implanted sample is annealed at various temperatures up to 1200 °C for 1 h in a nitrogen atmosphere.

### 3.2. Equipment

The main analytical techniques used in our laboratory are Raman, PL, and UV-visible absorption methods. Two setups have been used to record the Raman and PL spectra. The first setup consists of an argon-ion laser (488 nm, Melles Griot 543-AP-A01), a spectrometer (Acton SpectraPro 500I), and a charge-coupled device camera (Andor InstaSpec IV). The laser beam is focused with a lens to a spot with a diameter of ~40 μm. The second setup allows us to perform micro-Raman and micro-PL measurements. It consists of a confocal Raman microscope (LabRAM HR800, Horiba Jobin Yvon) equipped with an argon-ion laser (488 nm). The size of the laser spot in the microscopic measurements is down to ~1 μm. For both setups, the spectral resolution is ~2 cm^−1^ and 10 cm^−1^ for Raman and PL measurements, respectively. The Raman bands of the films annealed at ≥1000 °C are fitted with two or three Gaussians. The position of the Raman band of Si-nc is obtained from the position of the higher-frequency Gaussian (~518–530 cm^–1^). The PL spectra are corrected for the spectral sensitivity of the apparatus. The Raman and PL intensities are normalized by the effective thickness $\int_{0}^{d}e^{\left(-2ax\right)}dx$ and $\int_{0}^{d}e^{\left(-ax\right)}dx$, respectively, where the same absorption coefficients α is assumed for the laser and Raman light, the absorption for the PL is neglected, and *d* is the film thickness [55].

Laser annealing of free-standing films is performed using a TEM_00_ beam of an argon-ion laser (488 nm, power up to 110 mW at the sample) focused to spots with diameters ranging from ~1 to ~40 μm. The exposure time is mainly ~1 s, but 10-ms and 100-s exposures is also used in some experiments. Most of laser-annealing studies are performed in air; however, a number of measurements are done in O_2_, N_2_, and Ar atmospheres.

The absorption coefficient is obtained from the transmission and reflection spectra [152], recorded with a fiber-optics spectrometer (SD2000, Ocean Optics) and a broadband light source (DH-2000, Top Sensor Systems) or by measuring transmitted and reflected laser light using a power meter (NOVA II, OPHIR). The micro-transmission measurements of the 488-nm light are carried out in the LabRAM microscope with a large-area photodiode located behind the sample.

Additional measurements by other methods have been available from cooperation: XPS [115,116,117,118] including measurements with high spatial resolution [140], FTIR spectroscopy with high spatial resolution [140], TEM including bright-field scanning (BF), STEM and EFTEM [140,141], EELS [140], SEM and EDS [141,142], AFM [141,142], and *m*-line measurements [115]. The equipment used in these methods is described in the given references.

### 3.3. PL-Filtering Effect

**Figure 20:**
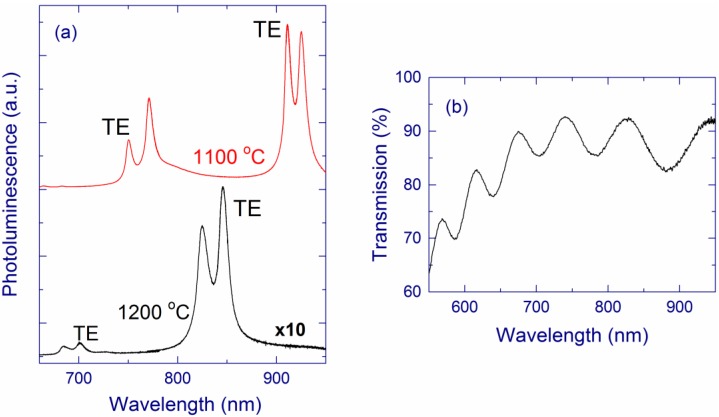
(**a**) Cut-off spectra of a SiO_1.5_ film annealed at 1100 and 1200 °C, measured from the sample edge along the film. The excitation spot was at a distance of several millimeters from the sample edge. The positions of the TE peaks are marked and the other bands have the TM polarization. The TE and TM peaks have polarization parallel and perpendicular to the film surface, respectively. (**b**) Transmission spectrum of the SiO_1.5_ film annealed at 1100 °C. Reproduced with permission from [115]. Copyright 2008, AIP Publishing LLC.

The method to measure the optical properties of the films utilizes the PL-filtering effect. The PL filtering is observed when the PL spectra are measured in a direction along the film surface. In this case, narrow and polarized spectral peaks are detected (Figure 20a). The PL filtering has been observed in various silica films containing Si-nc [49,57,59,79,85,86,87,94].

The PL-filtering effect can be interpreted in terms of delocalization of guided modes near the cut-off frequency [49,57,79,85]. The minimal losses occur near the cut-off wavelengths, for which the delocalized light travels mostly in the transparent substrate rather than in the absorbing film. Thus, the narrow peaks seen in the PL spectra obtained in the waveguiding direction correspond to the cut-off wavelengths. The mode localization is a function of the generalized frequency parameter written for an asymmetrical planar waveguide in the form:

(1) $$V=2\pi \sqrt{\left(n_{1}^{2}-n_{2}^{2}\right)}d/\text{λ}$$

where $n_{1}$ and $n_{2}$ are the refractive indexes of the film and substrate, respectively, *d* is the film thickness, and λ is the wavelength. This equation is obtained assuming weak-guiding approximation $\left(n_{1}-n_{2}\right)\ll 1$ [127]. For the cut-off condition V=(2m+1)π/2, Equation (1) yields the cut-off wavelengths.

Figure 20b shows a typical transmission spectrum of an annealed SiO_1.5_ film on a silica substrate. The spacing of the interference patterns (in cm^–1^) can be written as:

(2) $$\delta \nu =1/2n_{1}d$$

which provides the optical thickness (*nd*). The numerical information can be extracted from the transmission spectrum by fitting it, for example, with the following empirical function:

(3) $$T\left(\text{λ}\right)=P_{7}+P_{1}\left(1-\frac{P_{3}}{\text{λ}-P_{2}}\right)\left[1-P_{4}\sin\left(\frac{P_{5}}{\text{λ}}+P_{6}\right)\right]$$

where *T* is the transmittance, and *P_i_* are the fitting parameters [49]. In particular, the *P*_5_ parameter is connected with the period of the oscillations, *i.e*., with the optical thickness. The TE refractive index is relevant to transmission spectra. By using Equation (1) for the spacing between the TE peaks and Equation (2), the TE refractive index and the film thickness can be extracted [85]. Next, the obtained layer thickness together with the spacing between the TM peak positions is used to calculate the TM refractive index. The obtained thickness is also used to calculate the absorption (extinction) coefficient from the transmission and reflection measurements.

The model described above is called a *simple model* because it uses a few simplifications: (i) Equation (1) corresponds to the weak-guiding approximation $\left(n_{1}-n_{2}\right)\ll 1$; (ii) the procedure uses only the interval between the cut-off peaks disregarding their actual positions; (iii) the material dispersion is neglected; and (iv) a step-like profile is assumed for the refractive index.

**Figure 21:**
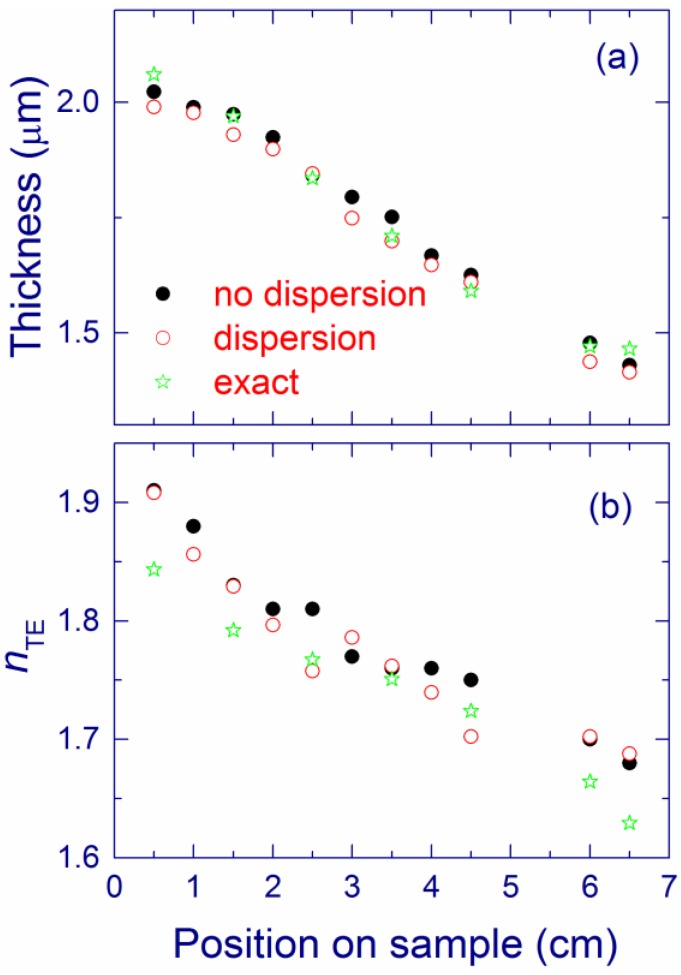
Optical parameters of a SiO*_x_* optical wedge (1.5 < *x* < 1.8) obtained with different models: simple model (solid circles), taking dispersion into account (open circles), and using the exact equations [153] (stars). Reproduced with permission from [115]. Copyright 2008, AIP Publishing LLC.

To take the dispersion of the material into account, the transmission spectra can be fitted with a function similar to Equation (3) but containing an additional term describing the wavelength dependence of the spacing δν [115]. Equations (1) and (2) are then solved taking the dispersion correction into account. The results show that the dispersion corrections for the refractive index and the film thickness are within experimental errors (Figure 21).

The refractive index and the film thickness can be also calculated using the model of reflection from a multilayer system [153], which does not limit the refractive index (exact equations). The analysis includes both spacing and positions of the cut-off peaks. Equation (3) with an additional term describing the material dispersion is also used in this case [115]. Again, the differences from the simple model are within the experimental errors (“exact” in Figure 21). Using the XPS data for a SiO_1.55_ film annealed at 1100 °C, it has been concluded that the use of smooth index profile of the refractive index is not crucial to obtain good description of the delocalized cut-off modes. These results suggest that the simple model is sufficient to achieve adequate estimates of the refractive index and film thickness. The *m-*line measurements lead to the results very similar to those obtained by the PL-filtering effect.

PL-filtering effect has also been interpreted in terms of substrate leaky (or radiation) modes of a planar waveguide [86,87]. In this model, the regular guided modes experience greater loss propagating over a macroscopic distance from the place of creation towards the sample edge. The most likely source of the loss is absorption and scattering by nanocrystals, as well as scattering at the “interface” between the core and cladding layers. In contrast, the beams responsible for generating the substrate modes travel shorter distances through the core region, and the substrate mode undergoes virtually no loss in traveling to the substrate facet. There seems to be no contradiction between the descriptions based on mode delocalization and substrate leaky modes. In particular, both of these models explain the PL-filtering effect based on a smaller absorption in the substrate compared to the film containing Si-nc.

### 3.4. X-Ray Photoelectron Spectroscopy

XPS measurements provide detailed information about the elemental and chemical composition of the material. Since only a thin layer of the sample (≤10 nm) is investigated with this method, the sample is sputtered with Ar^+^ ions to obtain information about the bulk material.

**Figure 22:**
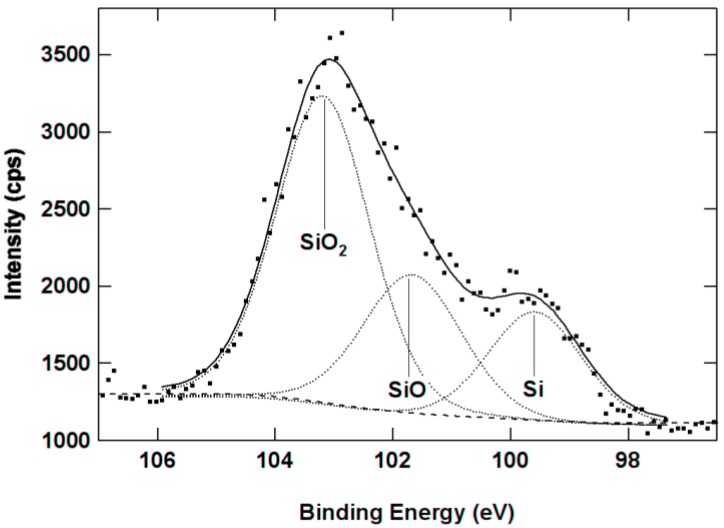
Typical XPS spectrum of a SiO*_x_* film fitted with three Gaussians corresponding to Si bonded as in elemental Si, SiO (mixture of suboxides), and SiO_2_. Figure courtesy of Jouko Lahtinen. Reproduced with permission from [115]. Copyright 2008, AIP Publishing LLC.

In the case of SiO*_x_* films, the Si 2*p* spectrum is fitted by several peaks to obtain the amounts of Si in different oxidation states. Three peaks are often used to describe Si atoms bonded as in crystalline Si (elemental Si), “SiO” (mixture of various suboxides), and SiO_2_ materials (Figure 22). More complicated fitting with five peaks can be used to obtain the five oxidation states of Si (elemental Si, Si_2_O, SiO, Si_2_O_3_, and SiO_2_) [154,155,156]. For our samples, the fittings with three and five peaks give similar amounts of elemental Si and SiO_2_ [116]. Moreover, the use of five fitting curves is problematic for low Si content.

### 3.5. Effective Medium Approximation

The effective medium approximation (Bruggeman theory) connects the effective dielectric constant with the dielectric constants of the three structural components and their volumetric fractions [157]:

(4) $$a\left(\frac{\varepsilon_{a}-\varepsilon_{m}}{\varepsilon_{a}+2\varepsilon_{m}}\right)+b\left(\frac{\varepsilon_{b}-\varepsilon_{m}}{\varepsilon_{b}+2\varepsilon_{m}}\right)+c\left(\frac{\varepsilon_{c}-\varepsilon_{m}}{\varepsilon_{c}+2\varepsilon_{m}}\right)=0$$

where *a*, *b*, and *c* are the volumetric fractions of each component, *ε_a,b,c_* are the corresponding dielectric constants, and *ε_m_* is the effective dielectric constant. The effective medium approximation is employed to calculate the complex refractive index n* = *n* + *ik* (where *k* is the extinction coefficient) using the chemical compositions (Si, SiO, and SiO_2_) obtained from the XPS measurements.

### 3.6. Temperature Measurements

**Figure 23:**
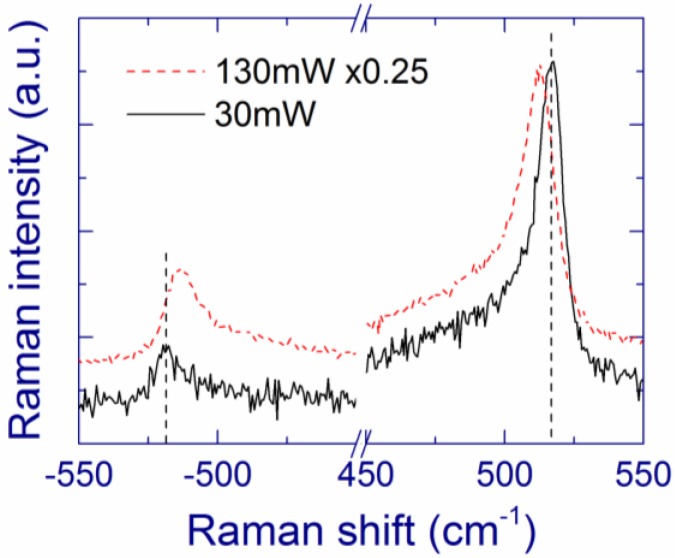
Raman spectra of a SiO_1.3_ film on a silica substrate measured with two laser powers (~30 and ~130 mW focused to a ~40 μm spot). The spectra are vertically shifted and scaled for better presentation.

Laser-induced temperature (*T*) can be measured *in-situ* using the intensities of the Stokes (*I*_S_) and anti-Stokes (*I*_AS_) Raman bands (Figure 23) and the relation:

(5) IASIS=Ae(−ERkT)

where *E*_R_ is the phonon energy and *A* is a correction coefficient [95,139]. The coefficient *A* ~ 0.95 is obtained by measuring the laser-induced temperature of a Si wafer as a function of the laser power and assuming room temperature in the irradiated volume at zero laser power.

### 3.7. Optical Absorption

The film absorption is obtained by measuring transmission and reflection in ultraviolet and visible regions using a broadband light source. The band gap of semiconductors can be obtained from the absorption spectra using the Tauc relation, according to which, the absorption spectrum obeys the following equation [88,158]:

(6) $$\sqrt{\text{α}\cdot h\nu }=A\left(h\nu -E_{Tauc}\right)$$

where *A* is a correction coefficient. Function $\sqrt{\alpha \cdot h\nu }$ is plotted *versus* hν and fitted with a straight line. The value of the optical gap (*E_Tauc_*) is obtained from the intercept of the linear fit with the abscissa.

## 4. Conclusions

Optical and structural properties of Si-nc in silica films have been described. In order to prepare Si-nc, SiO*_x_* (*x* < 2) films are annealed in a furnace at temperatures up to 1200 °C. For different Si contents, the Si-nc Raman signal and the absorption coefficient are proportional to the amount of elemental Si detected by XPS. On the other hand, the Raman scattering cross-section of elemental Si is about three times smaller than that of crystalline Si. This difference can be explained by the presence of small Si-nc (<2 nm) and/or by the different properties of bulk and nanoscale Si.

The measured optical properties of SiO*_x_* films are compared with the values estimated by the effective medium approximation using the XPS results. A good agreement is found between the measured and calculated refractive index. The results for absorption suggest high transparency of nanoscale suboxide in the annealed samples. Thermal annealing increases the degree of Si crystallization; however, the crystallization is not complete after annealing at 1200 °C. The extinction coefficient of elemental Si is found to be between the values of crystalline and amorphous Si.

The PL quantum yield increases as the amount of elemental Si decreases. It follows that the 1.5-eV PL from the SiO*_x_* films annealed above 1000 °C is probably not directly from Si-nc responsible for absorption and detected by Raman spectroscopy. According to these results, the light-emitting centers may be small (~1 nm) oxidized Si grains or oxygen-related defects, which are not detectable by Raman spectroscopy. This conclusion is in agreement with the theoretical simulations [114].

The as-prepared SiO*_x_* films deposited by MBD and ion implantation are structurally and optically very different. Relatively large amorphous clusters (>2 nm) are present in the MBD films whereas they are absent in the implanted samples as suggested by absorption and Raman spectroscopy. After annealing at ≥1100 °C, these two kinds of samples become similar and possess comparable amounts of Si-nc.

Laser-induced thermal effects are found for SiO*_x_* films on silica substrates when illuminated by focused laser light. In our experiments, a temperature of ~350 °C was estimated for SiO*_x_* films (*x* ~ 1.3) on a silica substrate for 488-nm laser intensity of ~10^4^ W cm^–2^, which decreases the Raman shift by ~6 cm^–1^. This effect should be taken into account in optical measurements using focused laser beams, especially in Raman microscopy.

CW laser light can produce very high temperatures in free-standing SiO*_x_* films and Si/SiO_2_ SLs, which strongly changes their structure and optical properties. The center of a laser-annealed area is very transparent and presumably consists of amorphous SiO_2_. Large Si-nc (up to 300 nm) are observed in the ring around the central region. These Si-nc possess high absorption of visible light and they are typically under compressive stress, which is connected with the crystallization from the liquid phase. Some of these large Si-nc exhibit surface features that are formed by eruption of pressurized Si from the film. Some of these “surface” Si-nc are removed from the film leading to holes of similar sizes. The presence of oxygen in the laser-annealing atmosphere decreases the amount of removed Si particles most probably due to oxidation of their surface.

The structure of laser-annealed areas is explained by thermodiffusion in temperature gradient, which means the macroscopic Si–SiO_2_ phase separation. Comparison of the structure of central regions for laser annealing in oxygen, air, and inert atmospheres excludes the dominating effect of Si oxidation, thus, supporting the mechanism of the macroscopic Si–SiO_2_ phase separation.

By using a strongly focused laser beam, the structural changes in the free-standing films can be made in areas of submicron sizes. A concept of high-density non-volatile optical memory with superior thermal stability is demonstrated where the information can be read, rewritten and erased by optical means.
